# Associations of non-traditional lipid-inflammatory parameters with all-cause mortality in middle-aged and older adults: Insights from two nationally representative cohorts

**DOI:** 10.1016/j.metop.2026.100507

**Published:** 2026-10-09

**Authors:** Xiaohang Yang, Luna Liu, Xiaoqian Du, Ling Gao, Yue Liu

**Affiliations:** a Shandong Clinical Research Center of Diabetes and Metabolic Diseases, Department of Endocrinology, Shandong Provincial Hospital Affiliated to Shandong First Medical University, Jinan, Shandong, 250021, China; b Key Laboratory of Endocrine Glucose & Lipids Metabolism and Brain Aging, Ministry of Education, Jinan, Shandong, 250021, China; c Shandong Institute of Endocrine and Metabolic Diseases, Jinan, Shandong, 250021, China; d "Chuangxin China" Innovation Base of Stem Cell and Gene Therapy for Endocrine Metabolic Diseases, Jinan, Shandong, 250021, China; e Shandong Engineering Laboratory of Prevention and Control for Endocrine and Metabolic Diseases, Jinan, Shandong, 250021, China

**Keywords:** Non-traditional lipid-inflammatory parameters, Lipid metabolism, Inflammation, Mortality

## Abstract

****Background**:**

Non-traditional lipid-inflammatory parameters are emerging biomarkers integrating lipid metabolism and inflammation. However, their association with all-cause mortality in middle-aged and older adults has not been systematically evaluated.

****Methods**:**

This study enrolled 9943 participants from the National Health and Nutrition Examination Survey (NHANES) and 9409 participants from the China Health and Retirement Longitudinal Study (CHARLS). Survey weights were applied to account for the complex sampling design. Kaplan–Meier analyses were performed to estimate cumulative survival probabilities. Cox proportional hazards models were used to examine the associations. Restricted cubic splines were fitted to explore potential dose–response relationships. Time-dependent ROC analyses were conducted to evaluate predictive performance at follow-up time points. DeLong tests were used to assess the statistical significance of differences in the areas under the curve. Calibration curves were constructed to assess the model calibration. Subgroup and sensitivity analyses were conducted to explore heterogeneity and assess the robustness of the results.

****Results**:**

Over a mean follow-up of 80.3 months in NHANES and 100.2 months in CHARLS, 2377 and 1246 deaths were recorded, respectively. Cumulative survival probabilities decreased across quartiles for all parameters except LCI-CRP (Log-rank *P* = 0.960) in NHANES. After adjusting for confounders, all parameters were positively associated with all-cause mortality in continuous analyses. In quartile analyses, the highest quartile carried a higher risk than the lowest, except for AIP-CRP (HR = 1.16, 95% CI: 0.88–1.52, *P* = 0.295) and LCI-CRP (HR = 1.28, 95% CI: 0.99–1.64, *P* = 0.056) in CHARLS. AIP-CRP exhibited a nonlinear dose–response relationship in both cohorts. The predictive performance of parameters improved with longer follow-up time in NHANES, whereas it fluctuated in CHARLS. DeLong tests showed that models incorporating parameters had better discriminative ability than the base model. Calibration curves indicated good calibration of the parameter-incorporating models. Hypertension significantly modified the associations of certain parameters in CHARLS. The results remained robust across all sensitivity analyses.

****Conclusions**:**

Non-traditional lipid-inflammatory parameters demonstrate significant independent associations with an elevated risk of all-cause mortality among middle-aged and older adults. Based on routine lipid and inflammatory tests, these parameters are simple, low-cost tools for early risk stratification.

## 1 Introduction

Global population aging is accelerating. The World Health Organization projects that the population aged 60 years and older will double by 2050 [1], while the United Nations reports that individuals aged 65 years and older are now the fastest-growing age group [2]. As a result of this demographic shift, deaths are increasingly concentrated among older adults. Specifically, annual deaths among individuals aged 70 years and older have more than doubled since 1990 [3]. Early identification of individuals at high risk of all-cause mortality is therefore essential for risk stratification and targeted prevention.

Dyslipidemia and chronic inflammation are well-established drivers of all-cause mortality [4], [5]. Traditional lipid parameters reflect the lipid profile and play a central role in cardiovascular risk assessment [6]. However, their utility in predicting all-cause mortality is limited by the “cholesterol paradox” [7], [8]. Non-traditional lipid parameters, such as non-high-density lipoprotein cholesterol (non-HDL-C) and remnant cholesterol (RC), capture residual risk of all-cause mortality missed by traditional parameters [9], [10]. Meanwhile, C-reactive protein (CRP), an inflammatory marker, independently predicts all-cause mortality risk [11], [12]. Notably, evidence suggests that co-elevation of RC and CRP is associated with a higher risk of all-cause mortality than either factor alone [13]. Thus, combined assessment of non-traditional lipid parameters and CRP may improve risk stratification for all-cause mortality.

Constructed by integrating non-traditional lipid parameters with CRP, non-traditional lipid-inflammatory parameters have been developed in recent research. Existing evidence has linked these parameters to cardiovascular outcomes, such as stroke and hypertension [14], [15]. However, their association with all-cause mortality remains unclear. This study aims to examine the association of non-traditional lipid-inflammatory parameters with all-cause mortality in middle-aged and older adults. The findings are expected to provide epidemiological evidence supporting the clinical application of combined lipid-inflammatory assessment strategies.

## 2 Methods

### 2.1 Study design and participants

This study utilized two nationally representative cohorts, the National Health and Nutrition Examination Survey (NHANES) and the China Health and Retirement Longitudinal Study (CHARLS). NHANES is a cross-sectional survey conducted by the National Center for Health Statistics (NCHS). It adopts a multistage, stratified probability sampling design to systematically assess the health and nutritional status of the general US population [16]. In this study, data from the 2005-2010 and 2015-2018 cycles were included to construct the baseline analytic population. CHARLS is a longitudinal cohort study organized by the National School of Development at Peking University. It aims to investigate the health determinants and their dynamic changes among Chinese adults aged 45 years and older [17]. The baseline survey (Wave 1) was initiated in 2011, and four waves of follow-up data have been released to date, namely Wave 2 (2013), Wave 3 (2015), Wave 4 (2018), and Wave 5 (2020). In this study, participants from Wave 1 were included to construct the baseline analytic population.

We then used uniform exclusion criteria for baseline participants in both cohorts. Details are provided in Fig. 1. Participants were sequentially excluded according to the following criteria: (1) Aged < 45 years; (2) Frailty status not assessable at baseline; (3) Missing measurements of total cholesterol (TC), triglyceride (TG), high-density lipoprotein cholesterol (HDL-C), LDL-C, or CRP; (4) Loss to follow-up; and (5) Missing sampling weight information. Finally, 9943 participants from NHANES and 9409 participants from CHARLS were included in the formal analyses.

**Fig. 1:**
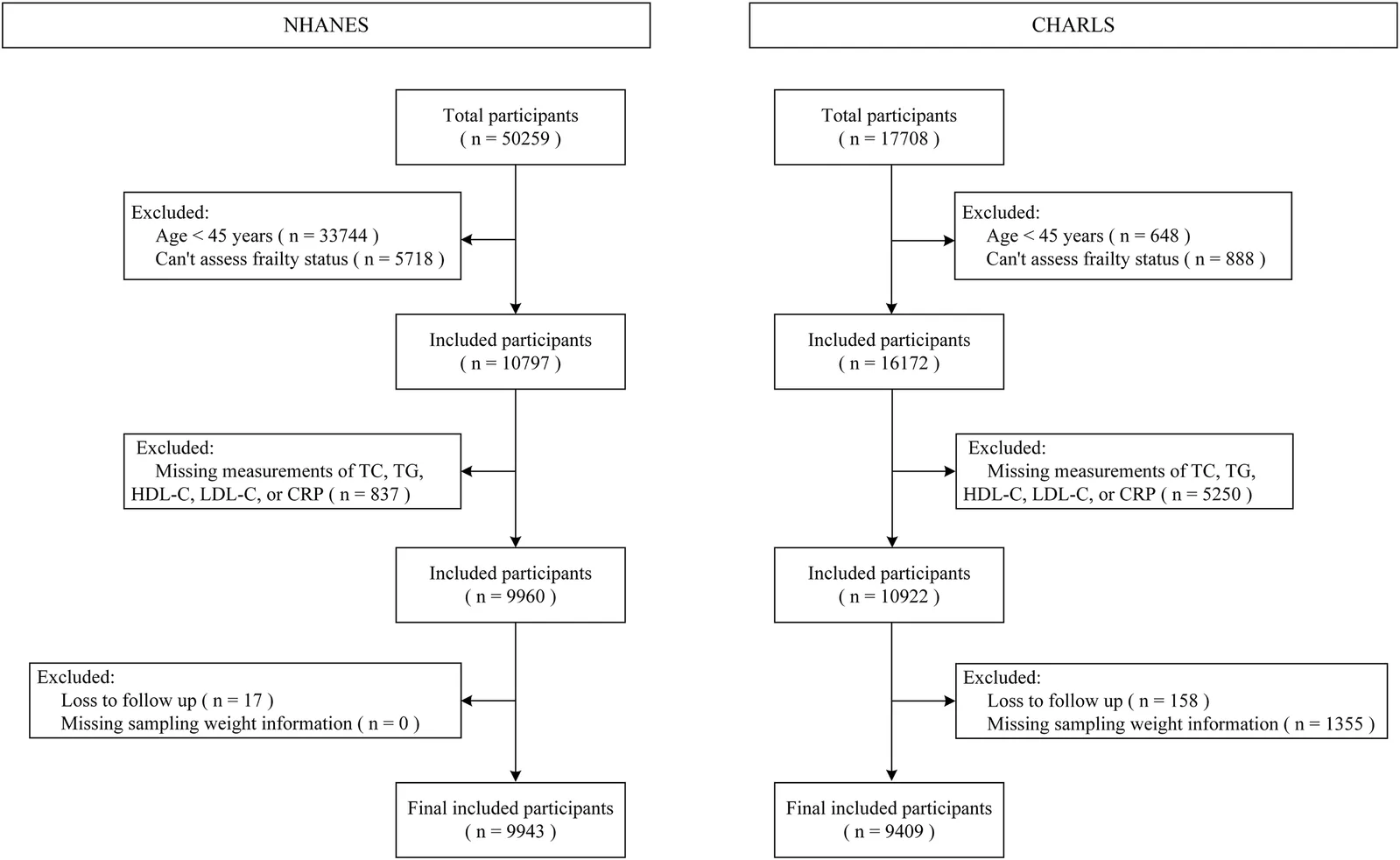
Flowchart of study participants. Abbreviations: NHANES, National Health and Nutrition Examination Survey; TC, total cholesterol; TG, triglyceride; HDL-C, high-density lipoprotein cholesterol; LDL-C, low-density lipoprotein cholesterol; CRP, C-reactive protein.

### 2.2 Exposures

The exposures of this study were eight non-traditional lipid-inflammatory parameters. These parameters were constructed by multiplying established non-traditional lipid parameters with CRP. The multiplicative formula enables composite parameters to simultaneously capture lipid metabolism and inflammatory status. During the baseline examination, fasting venous blood samples were collected from participants in both cohorts. Four traditional lipid parameters (TC, TG, HDL-C, and LDL-C) were measured using enzymatic methods and expressed in mg/dL. CRP was assessed using cohort- and cycle-specific immunoassays and expressed in mg/L [18], [19]. For participants with missing measurements in certain NHANES cycles, we used the Friedewald formula to estimate LDL-C [20]. Based on these laboratory measurements, the following eight non-traditional lipid parameters were first calculated, with the specific formulas presented below:

(1) non-HDL-C (mg/dL) = TC − HDL-C [21];
(2) RC (mg/dL) = TC − HDL-C − LDL-C [22];
(3) Atherogenic index of plasma (AIP) (log-transformed ratio) = lg (TG/HDL-C) [23];
(4) Atherogenic coefficient (AC) (ratio) = (TC – HDL-C)/HDL-C [24];
(5) Castelli's index-I (CRI-I) (ratio) = TC/HDL-C [25];
(6) Castelli's index-II (CRI-II) (ratio) = LDL-C/HDL-C [26];
(7) Lipoprotein combine index (LCI) [(mg/dL)^2^] = TC × TG × LDL-C/HDL-C [27];
(8) RC/HDL-C (ratio) = (TC – HDL-C – LDL-C)/HDL-C [14];

To maintain consistency with the units of traditional lipid parameters, CRP was converted to mg/dL by dividing by 10. Subsequently, eight non-traditional lipid-inflammatory parameters were calculated using the following formula:

Non-traditional lipid-inflammatory parameter = Non-traditional lipid parameter × CRP (mg/L)/10 [28].

### 2.3 Follow-up and outcome

The endpoint of this study was all-cause mortality, which is defined as death events caused by heart disease, cancer, and all other causes. In NHANES, mortality data were obtained by linking baseline survey data with death certificate records from the National Death Index. Survival status was determined from this linkage. Follow-up time was calculated as the interval from the baseline examination date to the date of death or the end of follow-up (December 31, 2019), whichever occurred first. Loss to follow-up was defined as the lack of valid mortality linkage data, indicated by ineligibility for follow-up, or missing/invalid person-months of follow-up. Participants who were still alive at the end of follow-up were censored [29]. In CHARLS, survival status was confirmed through standardized questionnaires at each follow-up wave [30]. Follow-up time was calculated from Wave 1. For participants with confirmed deaths, follow-up ended at the date of death. For those without reported deaths, follow-up ended at the last interview or the end of the study, whichever occurred first. Loss to follow-up was defined as the absence of any successful follow-up interview before the end of follow-up without a recorded death. These participants were censored at the date they were last known to be alive. The specific follow-up time distribution is detailed in Supplementary Table 1.

### 2.4 Covariates

Based on previous relevant studies and clinical relevance [31], [32], [33], [34], the covariates included in this study were age, sex, marital status, education, smoking status, drinking status, diabetes, hypertension, body mass index (BMI), and systolic blood pressure (SBP). These covariates were grouped into four categories: sociodemographic characteristics (age, sex, marital status and education); Lifestyle factors (smoking and drinking); Disease burden factors (diabetes and hypertension); Anthropometric measures (BMI and SBP). Considering the differences in data collection between two cohorts, education was categorized as less than high school, high school, and college or above in NHANES, and as less than primary school, primary school, junior high school, and high school or above in CHARLS.

### 2.5 Weights information

This study incorporated survey weights into the analyses. In NHANES, we first constructed multi-cycle weights suitable for this study (the 2-year sample weights divided by the number of included cycles). Stratification and clustering variables were simultaneously incorporated to ensure accurate effect estimation and standard error calculation. In CHARLS, we applied the biosample weight to account for individual nonresponse bias in biological sample collection. Post-stratification adjustment by age, sex, and residence further ensured that the sample represents the Chinese middle-aged and older population.

### 2.6 Statistical analysis

Covariates with fewer than 20% missing values were assumed to be missing at random. Missing values were imputed using multivariate imputation by chained equations (MICE) (Details in Supplementary Tables 2 and 3). Baseline characteristics were stratified by survival status. Continuous variables were presented as weighted medians with interquartile ranges (IQRs), and compared using weighted Wilcoxon rank-sum tests. Categorical variables were presented as unweighted counts and weighted percentages, and compared using weighted chi-square tests. Multicollinearity was assessed by calculating the variance inflation factors and Spearman correlation coefficients (Supplementary Figs. 1 and 2). Kaplan-Meier survival analyses were used to estimate cumulative survival probabilities, with comparisons performed using log-rank tests. Cox proportional hazards models were applied to examine the associations between eight non-traditional lipid-inflammatory parameters and all-cause mortality. Each parameter was included in the models both as a Z-standardized continuous variable and as a quartile-categorized variable. The data distribution characteristics of all parameters are summarized in Supplementary Table 4. Results were presented as hazard ratios (HRs) with corresponding 95% confidence intervals (CIs). The proportional hazards assumptions were examined using Schoenfeld tests (Supplementary Table 5). Three progressively adjusted models were constructed in this study: Model 1 was adjusted for age and sex; Model 2 was further adjusted for marital status, education, smoking, and drinking; and Model 3, as the final model, was additionally adjusted for diabetes, hypertension, BMI, and SBP. Restricted cubic spline (RCS) models were applied to explore the dose–response relationships. Results were presented as *P* values for overall association (*P-overall*) and nonlinearity (*P-nonlinear*). Time-dependent ROC analyses were conducted to evaluate the predictive performance of each parameter at the follow-up time points (1, 3, 5, and 7 years). The base prediction model included age, sex, marital status, education, smoking, drinking, diabetes, hypertension, BMI, and SBP. Each parameter was then added to the base model to assess the improvement in prediction performance. The areas under the curve (AUCs) and their 95% CIs were calculated using 1000 Bootstrap resamples. DeLong tests were used to evaluate the statistical significance of AUC differences (ΔAUC) of each parameter compared with the base model. Calibration curves were constructed to evaluate the agreement between predicted and observed risks. Subgroup analyses were conducted to examine potential effect heterogeneity. Stratification variables included age, sex, smoking, drinking, diabetes, hypertension, and frailty status. For each stratification variable, *P* values for interaction (*P* for interaction) were calculated to assess effect modification. Given the multiple comparisons involved, the Benjamini–Hochberg procedure was applied to control the false discovery rate, and adjusted *P* values for interaction [*P* for interaction (BH-FDR)] are reported. Frailty status was assessed based on the frailty index (FI) and categorized as non-frail (FI < 0.10), pre-frail (0.10 ≤ FI < 0.21), and frail (FI ≥ 0.21) [35], [36]. To assess the robustness of the results, several sensitivity analyses were performed. Given the parallel analytical framework, the main analyses were restricted to covariates available in both cohorts. As a supplement, race and poverty income ratio (PIR) were additionally adjusted for in NHANES, while residence was further adjusted for in CHARLS. In addition, major clinical comorbidities, including heart disease, stroke, and cancer, were further adjusted for to account for potential confounding from baseline disease status. To minimize potential reverse causation bias, analyses were repeated after excluding participants who died within the first year of follow-up. Considering that lipid-lowering medications may directly affect lipid parameter levels, further verification was performed after excluding participants taking lipid-lowering medications. Finally, complete-case analyses were conducted to examine the influence of the imputation strategy on the results.

All statistical analyses were performed in R software (version 4.4.2). Hypothesis tests were two-sided, with statistical significance defined as *P* < 0.05.

## 3 Results

### 3.1 Baseline characteristics of the study cohorts

Final analyses enrolled 9943 participants from NHANES and 9409 participants from CHARLS. Over a mean follow-up of 80.3 months in NHANES and 100.2 months in CHARLS, 2377 and 1246 deaths were recorded, respectively. Specific event distribution during the follow-up period is presented in Supplementary Figure 3. Table 1 presents the weighted baseline characteristics of the overall study population and those stratified by survival status. Compared with survivors, non-survivors were older, more likely to be male and less educated, and more frequently had other marital statuses and smoking habits. They also exhibited greater frailty severity, higher levels of SBP, CRP, and all eight non-traditional lipid-inflammatory parameters, but lower levels of BMI, TC, and LDL-C. Furthermore, non-survivors bore a heavier disease burden, with higher prevalences of diabetes and hypertension. All the above comparisons reached statistical significance (*P* < 0.05).

**Table 1:** Weighted baseline characteristics of participants according to survival status.

Variables	NHANES^a^				CHARLS^b^			
	Overall	Survivor	Non-Survivor	*P*	Overall	Survivor	Non-Survivor	*P*
**Sample**	9943	7566	2377		9409	8163	1246	
**Age, years**	66.00 (60.00, 74.00)	65.00 (60.00, 71.00)	74.00 (65.00, 80.00)	<0.001	58.00 (52.00, 66.00)	57.00 (51.00, 64.00)	71.00 (63.00, 78.00)	<0.001
**Sex, n(%)**				<0.001				<0.001
Male	4926 (45%)	3536 (44%)	1390 (51%)		4370 (47.3%)	3616 (45.6%)	754 (58.4%)	
Female	5017 (55%)	4030 (56%)	987 (49%)		5039 (52.7%)	4547 (54.4%)	492 (41.6%)	
**Marital status, n(%)**				<0.001				<0.001
Married	5427 (60%)	4297 (63%)	1130 (50%)		8208 (85.8%)	7306 (88.8%)	902 (66.5%)	
Other	4516 (40%)	3269 (37%)	1247 (50%)		1201 (14.2%)	857 (11.2%)	344 (33.5%)	
**Education, n(%)**				<0.001				<0.001
Less than high school^a^/Less than primary school^b^	3088 (19%)	2187 (16%)	901 (31%)		4610 (44.5%)	3832 (41.7%)	778 (63.2%)	
High school^a^/Primary school^b^	2410 (27%)	1798 (26%)	612 (28%)		2041 (21.5%)	1776 (21.5%)	265 (21.0%)	
College or above^a^/Junior high school^b^	4445 (54%)	3581 (57%)	864 (41%)		1850 (22.1%)	1704 (23.7%)	146 (10.7%)	
High school or above^b^	-	-	-		908 (11.9%)	851 (13.1%)	57 (5.1%)	
**Smoking, n(%)**				<0.001				<0.001
Never	4684 (48%)	3775 (50%)	909 (38%)		5736 (61.1%)	5152 (63.0%)	584 (48.1%)	
Former	3592 (36%)	2561 (35%)	1031 (43%)		832 (9.6%)	645 (8.5%)	187 (17.0%)	
Current	1667 (16%)	1230 (15%)	437 (19%)		2841 (29.3%)	2366 (28.4%)	475 (34.9%)	
**Drinking, n(%)**				<0.001				<0.001
Never	2923 (25%)	2242 (24%)	681 (28%)		5747 (60.8%)	5083 (61.6%)	664 (54.9%)	
Former	1954 (19%)	1293 (17%)	661 (27%)		806 (8.0%)	635 (7.4%)	171 (12.1%)	
Current	5066 (56%)	4031 (59%)	1035 (45%)		2856 (31.3%)	2445 (31.0%)	411 (33.0%)	
**Diabetes, n(%)**				<0.001				<0.001
No	7627 (81%)	5875 (83%)	1752 (76%)		7989 (84.1%)	7004 (85.2%)	985 (76.7%)	
Yes	2316 (19%)	1691 (17%)	625 (24%)		1420 (15.9%)	1159 (14.8%)	261 (23.3%)	
**Hypertension, n(%)**				<0.001				<0.001
No	4099 (45%)	3246 (47%)	853 (37%)		4838 (49.4%)	4356 (51.5%)	482 (35.6%)	
Yes	5844 (55%)	4320 (53%)	1524 (63%)		4571 (50.6%)	3807 (48.5%)	764 (64.4%)	
**Frailty status, n(%)**				<0.001				<0.001
Non-frail	2022 (23%)	1708 (25%)	314 (13%)		4192 (46.9%)	3802 (49.5%)	390 (30.1%)	
Pre-frail	4087 (43%)	3245 (45%)	842 (35%)		3830 (39.2%)	3342 (39.7%)	488 (36.2%)	
Frail	3834 (34%)	2613 (30%)	1221 (52%)		1387 (13.8%)	1019 (10.8%)	368 (33.7%)	
**BMI, kg/m^2^**	28.60 (25.03, 33.07)	28.80 (25.20, 33.35)	27.78 (24.00, 31.72)	<0.001	23.30 (21.03, 25.98)	23.45 (21.22, 26.10)	22.33 (19.84, 25.04)	<0.001
**SBP, mmHg**	128.67 (117.33, 142.67)	128.00 (116.67, 141.33)	133.00 (118.67, 148.00)	<0.001	127.00 (114.50, 142.50)	126.00 (114.00, 141.00)	137.50 (121.00, 153.00)	<0.001
**TC, mg/dL**	193.00 (166.00, 223.00)	196.00 (169.00, 224.00)	185.00 (158.00, 216.00)	<0.001	189.43 (166.24, 214.18)	189.82 (167.40, 214.56)	184.41 (160.44, 213.40)	0.018
**TG, mg/dL**	128.86 (89.00, 187.89)	128.86 (89.00, 187.89)	128.86 (89.00, 186.00)	0.781	107.08 (76.11, 158.41)	107.08 (76.11, 159.30)	105.32 (74.34, 153.99)	0.209
**HDL-C, mg/dL**	53.00 (43.00, 66.00)	53.00 (43.00, 66.00)	51.00 (42.00, 64.00)	0.003	48.33 (39.43, 58.76)	47.94 (39.43, 58.38)	49.48 (39.05, 61.08)	0.076
**LDL-C, mg/dL**	109.00 (85.00, 134.00)	111.00 (86.62, 135.00)	100.00 (78.00, 127.00)	<0.001	113.27 (93.17, 136.47)	114.05 (93.94, 136.86)	108.63 (86.99, 133.38)	<0.001
**CRP, mg/L**	2.17 (1.00, 4.81)	2.10 (0.91, 4.60)	2.60 (1.10, 6.10)	<0.001	1.08 (0.56, 2.24)	1.03 (0.54, 2.11)	1.48 (0.76, 3.87)	<0.001
**non-HDL-C-CRP**	29.30 (12.43, 69.27)	28.60 (12.10, 66.34)	33.60 (13.97, 80.22)	<0.001	15.31 (7.34, 33.09)	14.50 (7.09, 31.24)	19.54 (9.85, 55.34)	<0.001
**RC-CRP**	5.67 (2.20, 14.88)	5.43 (2.09, 14.05)	6.75 (2.60, 18.07)	<0.001	2.23 (0.85, 6.08)	2.12 (0.82, 5.72)	3.04 (1.15, 9.72)	<0.001
**AIP-CRP**	0.07 (0.02, 0.21)	0.07 (0.02, 0.20)	0.08 (0.02, 0.25)	<0.001	0.03 (0.01, 0.10)	0.03 (0.01, 0.09)	0.04 (0.01, 0.13)	0.010
**AC-CRP**	0.56 (0.22, 1.41)	0.53 (0.22, 1.35)	0.64 (0.24, 1.65)	<0.001	0.32 (0.14, 0.75)	0.30 (0.14, 0.72)	0.45 (0.19, 1.15)	<0.001
**CRI-I-CRP**	0.78 (0.33, 1.90)	0.75 (0.32, 1.80)	0.92 (0.37, 2.25)	<0.001	0.44 (0.20, 0.99)	0.42 (0.20, 0.94)	0.59 (0.27, 1.63)	<0.001
**CRI-II-CRP**	0.43 (0.17, 1.10)	0.42 (0.17, 1.06)	0.49 (0.19, 1.29)	<0.001	0.25 (0.12, 0.58)	0.24 (0.12, 0.56)	0.36 (0.16, 0.88)	<0.001
**LCI-CRP**	10,938.53 (3514.49, 33,982.39)	10,762.76 (3459.46, 33,124.93)	11,578.43 (3879.83, 36,850.00)	0.029	5697.69 (2014.01, 16,370.79)	5487.80 (1972.41, 15,725.08)	7364.68 (2557.01, 22,896.75)	<0.001
**RC/HDL-C-CRP**	0.11 (0.04, 0.30)	0.10 (0.04, 0.29)	0.13 (0.05, 0.38)	<0.001	0.05 (0.02, 0.14)	0.04 (0.02, 0.13)	0.07 (0.02, 0.19)	<0.001

Notes: Continuous variables were presented as weighted medians (Q1, Q3), and compared using weighted Wilcoxon rank-sum tests. Categorical variables were presented as unweighted counts and weighted percentages, and compared using weighted chi-square tests.

Abbreviations: NHANES, National Health and Nutrition Examination Survey; CHARLS, China Health and Retirement Longitudinal Study; BMI, body mass index; SBP, systolic blood pressure; TC, total cholesterol; TG, triglyceride; HDL-C, high-density lipoprotein cholesterol; LDL-C, low-density lipoprotein cholesterol; CRP, C-reactive protein.

a Education levels for NHANES participants.

b Education levels for CHARLS participants.

### 3.2 Associations of non-traditional lipid-inflammatory parameters with all-cause mortality

Weighted Kaplan-Meier survival curves revealed a decreasing trend in cumulative survival across quartiles. In NHANES, the cumulative survival rate in the highest quartile (Q4) was lower than that in the lowest quartile (Q1) for all parameters except LCI-CRP (Log-rank *P* = 0.960). In CHARLS, all parameters demonstrated statistically significant between-group differences in survival curves (Log-rank *P* < 0.01) (Supplementary Figs. 4 and 5). Table 2 and Supplementary Table 6 present the results of weighted Cox proportional hazards regression models with stepwise adjustment for covariates. In final models, all parameters treated as continuous variables were positively associated with all-cause mortality in both cohorts (*P* < 0.001). However, cohort-specific differences emerged when parameters were categorized into quartiles. In NHANES, Q4 was significantly associated with higher all-cause mortality risk compared with Q1 across all parameters (*P* < 0.01), with statistically significant trend tests (*P*_*trend*_ < 0.001). CRI-I-CRP exhibited the strongest association (HR = 1.59, 95% CI: 1.40–1.81, *P* < 0.001). In CHARLS, neither AIP-CRP (HR = 1.16, 95% CI: 0.88–1.52) nor LCI-CRP (HR = 1.28, 95% CI: 0.99–1.64) showed significant associations with all-cause mortality (*P* > 0.05), and the trend test for AIP-CRP was non-significant (*P* _*trend*_ = 0.204).

**Table 2:** Association of non-traditional lipid-inflammatory parameters with all-cause mortality.

Parameters	NHANES		CHARLS	
	HR (95% CI)	*P*	HR (95% CI)	*P*
**non-HDL-C-CRP**				
Per 1-SD	1.22 (1.16-1.29)	<0.001	1.15 (1.09-1.22)	<0.001
Q1	Ref		Ref	
Q2	1.13 (0.99-1.28)	0.064	1.04 (0.82-1.33)	0.723
Q3	1.22 (1.04-1.42)	0.012	1.38 (1.06-1.80)	0.017
Q4	1.56 (1.36-1.78)	<0.001	1.54 (1.22-1.95)	<0.001
***P*_*trend*_**	<0.001		<0.001	
**RC-CRP**				
Per 1-SD	1.21 (1.15-1.27)	<0.001	1.15 (1.08-1.22)	<0.001
Q1	Ref		Ref	
Q2	1.05 (0.89-1.25)	0.563	1.11 (0.87-1.40)	0.398
Q3	1.14 (1.00-1.31)	0.056	1.41 (1.09-1.83)	0.009
Q4	1.45 (1.27-1.64)	<0.001	1.58 (1.21-2.08)	<0.001
***P*_*trend*_**	<0.001		<0.001	
**AIP-CRP**				
Per 1-SD	1.21 (1.15-1.28)	<0.001	1.13 (1.06-1.20)	<0.001
Q1	Ref		Ref	
Q2	0.84 (0.73-0.97)	0.015	0.89 (0.71-1.13)	0.340
Q3	0.93 (0.82-1.06)	0.277	0.96 (0.75-1.23)	0.753
Q4	1.22 (1.07-1.39)	0.002	1.16 (0.88-1.52)	0.295
***P*_*trend*_**	<0.001		0.204	
**AC-CRP**				
Per 1-SD	1.23 (1.17-1.30)	<0.001	1.14 (1.07-1.20)	<0.001
Q1	Ref		Ref	
Q2	1.05 (0.93-1.19)	0.393	1.07 (0.84-1.37)	0.557
Q3	1.21 (1.04-1.41)	0.015	1.24 (0.94-1.64)	0.124
Q4	1.49 (1.32-1.69)	<0.001	1.47 (1.13-1.90)	0.004
***P*_*trend*_**	<0.001		0.001	
**CRI-I-CRP**				
Per 1-SD	1.25 (1.19-1.31)	<0.001	1.15 (1.08-1.21)	<0.001
Q1	Ref		Ref	
Q2	1.13 (0.99-1.29)	0.071	1.13 (0.90-1.42)	0.287
Q3	1.22 (1.04-1.43)	0.014	1.31 (1.00-1.71)	0.051
Q4	1.59 (1.40-1.81)	<0.001	1.59 (1.24-2.05)	<0.001
***P*_*trend*_**	<0.001		<0.001	
**CRI-II-CRP**				
Per 1-SD	1.23 (1.17-1.30)	<0.001	1.13 (1.07-1.20)	<0.001
Q1	Ref		Ref	
Q2	1.12 (0.99-1.26)	0.068	1.05 (0.82-1.33)	0.705
Q3	1.20 (1.02-1.41)	0.029	1.31 (0.99-1.72)	0.055
Q4	1.52 (1.34-1.73)	<0.001	1.44 (1.13-1.84)	0.003
***P*_*trend*_**	<0.001		<0.001	
**LCI-CRP**				
Per 1-SD	1.17 (1.10-1.24)	<0.001	1.12 (1.06-1.19)	<0.001
Q1	Ref		Ref	
Q2	1.02 (0.92-1.15)	0.668	0.89 (0.71-1.12)	0.321
Q3	1.10 (0.98-1.24)	0.115	1.01 (0.77-1.34)	0.922
Q4	1.22 (1.09-1.37)	<0.001	1.28 (0.99-1.64)	0.056
***P*_*trend*_**	<0.001		0.025	
**RC/HDL-C-CRP**				
Per 1-SD	1.21 (1.15-1.28)	<0.001	1.14 (1.07-1.21)	<0.001
Q1	Ref		Ref	
Q2	0.99 (0.86-1.14)	0.851	1.15 (0.91-1.45)	0.241
Q3	1.09 (0.96-1.25)	0.192	1.32 (1.00-1.74)	0.049
Q4	1.37 (1.21-1.55)	<0.001	1.50 (1.14-1.97)	0.004
***P*_*trend*_**	<0.001		0.003	

Note: Models were adjusted for age, sex, marital status, education, smoking, drinking, diabetes, hypertension, BMI, and SBP.

Abbreviations: NHANES, National Health and Nutrition Examination Survey; CHARLS, China Health and Retirement Longitudinal Study; BMI, body mass index; SBP, systolic blood pressure; HR, hazard ratio; CI, confidence interval; Ref, reference.

### 3.3 Potential dose–response relationships

Weighted RCS analyses revealed the potential dose–response relationships. As shown in Fig. 2 and Supplementary Figure 6, significant overall associations were observed between all parameters and all-cause mortality (*P-overall* < 0.01). Among them, AIP-CRP exhibited a significant nonlinear dose–response relationship in both cohorts (*P-nonlinear* < 0.001), whereas the remaining parameters showed linear associations (*P-nonlinear* > 0.05).

**Fig. 2:**
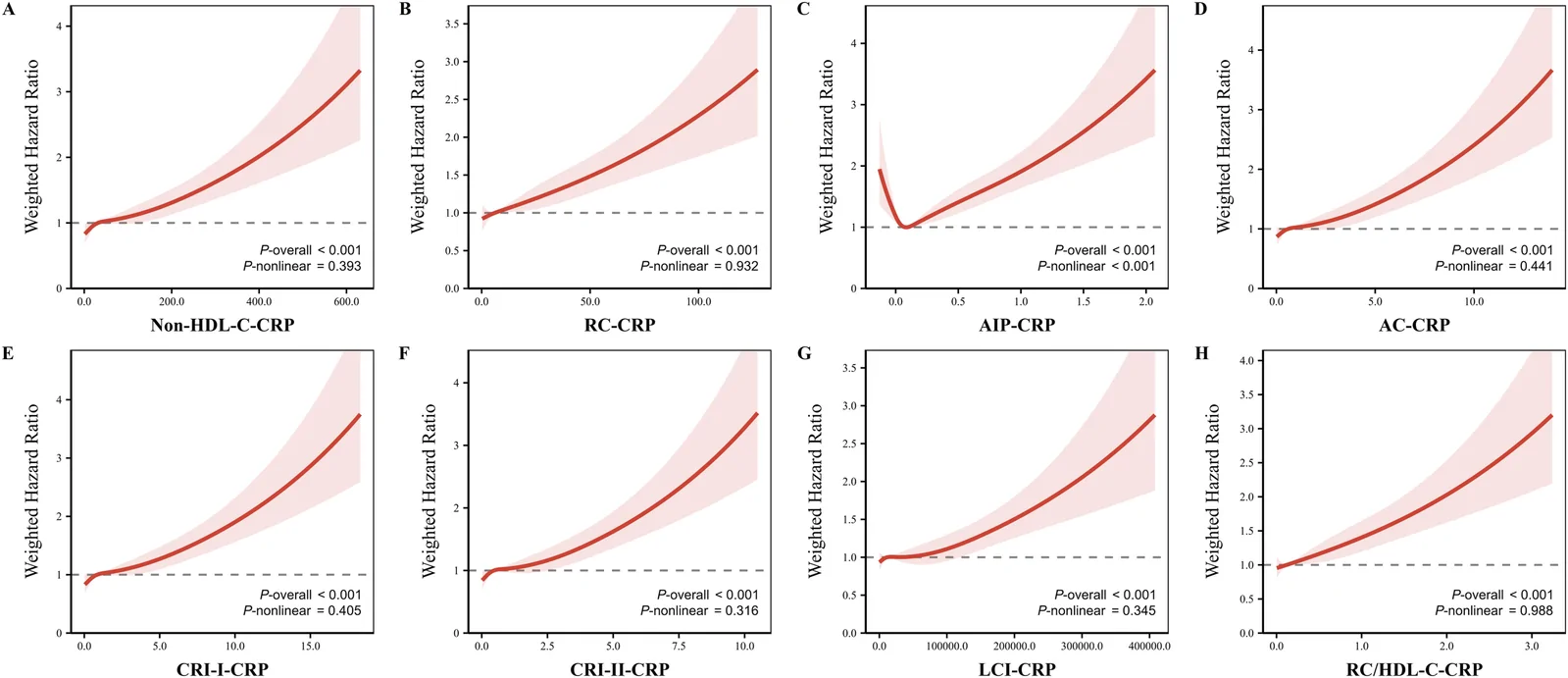
Weighted RCS analyses of the non-traditional lipid-inflammatory parameters with all-cause mortality in NHANES. Note: Models were adjusted for age, sex, marital status, education, smoking, drinking, diabetes, hypertension, BMI, and SBP. Abbreviations: NHANES, National Health and Nutrition Examination Survey.

### 3.4 Predictive performance

Time-dependent ROC curve analyses were used to evaluate predictive performance. In NHANES, discriminative ability increased progressively with longer follow-up. CRI-I-CRP showed relatively strong discrimination across most time points. In CHARLS, discriminative ability fluctuated over the follow-up period. non-HDL-C-CRP showed relatively strong discrimination in the short term (1–3 years), whereas RC-CRP was more favorable for long-term prediction (5–7 years) (Fig. 3 and Supplementary Table 7). DeLong tests showed that models incorporating these parameters had better discriminative ability than the base model (Supplementary Table 8). Calibration curves further indicated good calibration of the parameter-incorporating models (Supplementary Fig. 7 and Supplementary Table 9).

**Fig. 3:**
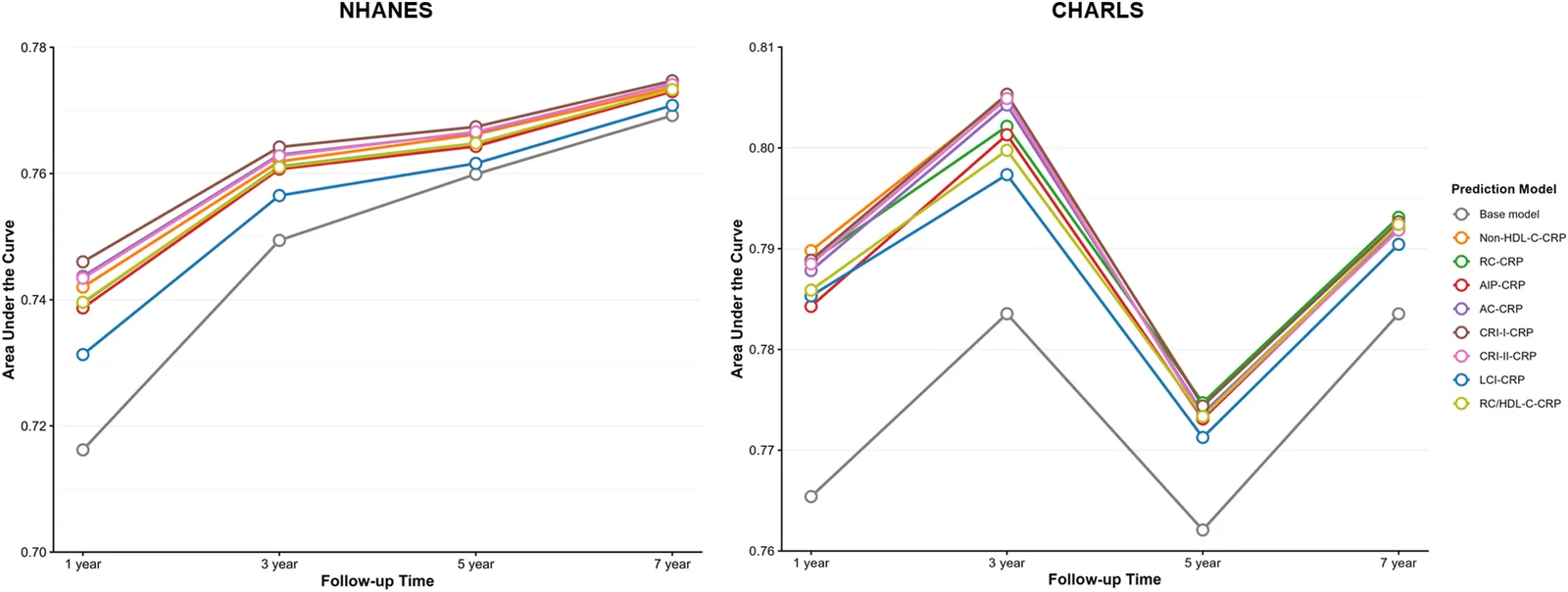
Predictive performance of non-traditional lipid-inflammation parameters at different follow-up time points. Note: Base model included age, sex, marital status, education, smoking, drinking, diabetes, hypertension, BMI, and SBP.

### 3.5 Subgroup analyses

Results of subgroup analyses were as follows. In NHANES, no significant effect modification was observed in the predefined subgroups [*P* for interaction (BH-FDR) > 0.05] (Supplementary Table 10). In CHARLS, hypertension significantly modified the associations of non-HDL-C–CRP, AC-CRP, CRI-I-CRP, and CRI-II-CRP with all-cause mortality. These associations appeared stronger in participants without hypertension [*P* for interaction (BH-FDR) < 0.01] (Supplementary Table 11).

### 3.6 Sensitivity analyses

To verify the robustness of our findings, we conducted several sensitivity analyses. First, we adjusted for cohort-specific confounders: race and PIR in NHANES, and residence in CHARLS. Second, we additionally adjusted for major clinical comorbidities (heart disease, stroke, and cancer) in a stepwise manner to examine potential confounding effects. Third, we excluded participants who died within the first year of follow-up to minimize potential reverse causation. Fourth, we excluded participants who reported taking lipid-lowering medications at baseline to avoid medication confounding. Finally, we repeated all analyses using complete-case data. Across all sensitivity analyses, the associations remained robust and were consistent with the main findings (Supplementary Tables 12–16).

## 4 Discussion

Using data from two nationally representative cohorts, this study evaluated the associations of eight non-traditional lipid-inflammatory parameters with all-cause mortality in middle-aged and older adults. We observed several key findings. First, when analyzed as continuous variables, all parameters were positively associated with all-cause mortality in both cohorts; however, when categorized into quartiles, AIP-CRP and LCI-CRP showed non-significant associations in CHARLS. Second, AIP-CRP showed a significant nonlinear dose–response relationship in both cohorts. Third, the predictive performance of these parameters improved progressively with longer follow-up in NHANES, whereas it fluctuated in CHARLS. Specifically, CRI-I-CRP maintained relatively strong discrimination across all follow-up time points in NHANES, while in CHARLS, non-HDL-C–CRP showed relatively strong discrimination in the short term and RC-CRP in the long term. Finally, hypertension significantly modified some of these associations in CHARLS.

Our findings align with and extend previous evidence. In a Danish cohort study involving over 300,000 participants, elevated RC was associated with a higher all-cause mortality risk [10]. Our results suggest that this association persists when RC interacts with CRP. The mathematical logic used to construct parameters is the same as the RCII proposed by Wang et al. [33]. Our study further confirmed the association between eight parameters including RCII (RC-CRP) and all-cause mortality. Together, evidence collectively supports the value of combined lipid–inflammatory assessment in risk stratification of all-cause mortality.

There are two notable exceptions in the CHARLS study: neither the AIP–CRP nor the LCI–CRP association reached statistical significance in Q4. A previous NHANES study showed similar findings to ours. They found no significant association between AIP and all-cause mortality across quartiles but a significant non-linear, U-shaped association [31]. Together with the non-linear association identified in our study, this suggests that quartile-based classification of AIP and its related parameters may mask its true risk associations. Population heterogeneity between the two cohorts offers a further explanation. The weighted baseline analysis revealed appreciable differences in lipid profiles between NHANES and CHARLS participants. Such distributional differences may alter the clinical relevance of the extreme quartiles for these two parameters. Differences in lipid-lowering therapy reinforce this concern. Statin use is considerably less prevalent in China than in the United States [37], which may yield distinct spectra of residual lipid-related risk and thereby modify the predictive utility of these composite indices. Dietary patterns may contribute as well. Traditional Chinese dietary patterns are typically higher in refined carbohydrates, which contrasts with Western patterns characterized by higher intakes of saturated fat and processed meat [38]. This contrast potentially shaped divergent lipid metabolic and inflammatory profiles. Consistent with this population-level variation, the PURE study across 22 countries demonstrated that the association of the TyG index with cardiovascular outcomes and mortality was evident in low- and middle-income countries but not in high-income countries [39]. Finally, the statistical characteristics of LCI may have played a role. As a composite of four traditional lipid parameters, LCI is sensitive to extreme values [40]. Previous studies have shown its instability in mortality prediction [41], [42]. This instability may be amplified after interacting with CRP, weakening its performance under quartile classification.

The predictive value of these parameters was time-dependent, but the pattern differed between cohorts. In NHANES, discrimination improved with longer follow-up time, whereas it fluctuated in CHARLS, with a decline at the 5-year time point. As shown in the event distribution plot, cumulative event curve under NHANES's mortality database–linked follow-up was markedly smoother than under CHARLS's wave-based follow-up, in which events are captured only at discrete survey waves. Such structural differences in follow-up design may partly account for the CHARLS fluctuation, as uneven event accrual between waves can destabilize time-dependent AUC estimates. That said, the point estimates of the 5-year AUCs fell within the CIs of the corresponding estimates at adjacent time points. This suggests that the apparent decline may reflect statistical uncertainty rather than a genuine loss of predictive performance. Beyond this fluctuation, the parameter with relatively strong discrimination in CHARLS also shifted over the follow-up period. A similar pattern has been reported for TyG–inflammation indices in a NHANES cohort. This study indicated that the optimal predictor changed from TyG-SIRI at 1 year to TyG-MLR at longer follow-up periods [43]. Taken together, these observations suggest that the predictive performance of parameters is cohort- and time-dependent. Accordingly, rankings based on a single time point should be interpreted with caution, and the performance of these parameters warrants validation in other Chinese aging cohorts.

These parameters capture a pathological process in which dyslipidemia and chronic inflammation amplify each other. On the one hand, lipid components can act as drivers of inflammation. Triglyceride-rich lipoproteins and their remnant particles can cross the arterial intima and be taken up by macrophages. This will lead to cholesterol deposition within the vessel wall and a local low-grade inflammatory response [44], [45]. Consistent with this direction of causality, Mendelian randomization evidence indicates that each 1 mmol/L higher RC level was causally associated with a 28% higher CRP level [46]. On the other hand, inflammation can alter the physiological functions of lipid components. Systemic and vascular inflammation can lead to high-density lipoprotein dysfunction. Specifically, it manifests as impaired reverse cholesterol transport, as well as reduced anti-inflammatory and antioxidant capabilities [47]. These reciprocal mechanisms may partly underlie the non-linear association of AIP-CRP observed in both cohorts. The ascending right arm of the curve plausibly reflects the superimposed effects of atherogenic dyslipidemia and systemic inflammation, which act synergistically to promote endothelial dysfunction and plaque formation. Conversely, the elevated risk at the low end of the distribution (the left arm of the curve) may reflect malnutrition, frailty, or occult chronic disease, in which both lipid and inflammatory reserves are depleted. Nevertheless, these mechanisms cannot fully explain the significant modifying effect of hypertension. Continuous mechanical stress on vascular endothelium in hypertension can induce endothelial inflammatory activation and cause synergistic injury with lipid deposition [48]. One would therefore expect the associations of these parameters to be more pronounced among individuals with hypertension; yet our results showed the opposite pattern. This paradox may reflect the dominance of blood pressure–driven injury in hypertensive individuals, which leaves less incremental predictive value for these parameters. In practice, these findings underscore the importance of considering hypertensive status when applying these parameters, while mechanistic studies are warranted to clarify the basis of this subgroup difference.

These parameters can be calculated directly from routine lipid testing combined with CRP measurement. In clinical practice, indicators should be selected according to the characteristics of the target population, the prediction time window, and the underlying disease status of patients. At the public health level, they provide simple, low-cost tools for large-scale all-cause mortality risk stratification, especially in resource-limited settings. This study has several strengths. The dual-cohort design allowed cross-validation of the findings in two national populations. The results were extrapolated to the national level by introducing survey weights. In addition, we compared eight parameters within a unified analytical framework. It combined dose–response analyses with dynamic prediction, and systematically assessed robustness and heterogeneity. Despite these strengths, we acknowledge several limitations of this study. First, the study design is inherently observational. Residual confounding cannot be excluded despite adjustment for multiple confounders. Second, these parameters were derived solely from single baseline measurements, which precludes capturing their dynamic trajectories during follow-up or the impact of therapeutic interventions. Future studies could leverage multi-wave follow-up data to conduct trajectory analyses. Third, LDL-C in certain NHANES cycles was estimated using the Friedewald formula, which may have affected the accuracy. Fourth, there were objective differences between the two cohorts in death confirmation, follow-up mode, and baseline medication recording. Finally, our results may have been influenced by participation bias, which is common in population-based studies.

## 5 Conclusion

This study demonstrates that non-traditional lipid-inflammatory parameters are positively associated with all-cause mortality among middle-aged and older adults. Requiring only routine lipid profiling and inflammatory testing, these parameters are simple, low-cost, and readily accessible as practical tools for the early risk stratification of all-cause mortality.

## CRediT authorship contribution statement

**Xiaohang Yang:** Writing – original draft, Investigation, Formal analysis, Data curation. **Luna Liu:** Visualization, Validation, Methodology. **Xiaoqian Du:** Validation, Resources. **Ling Gao:** Writing – review & editing, Supervision. **Yue Liu:** Writing – review & editing, Supervision, Project administration, Conceptualization.

## Data availability statement

The data that support the findings of this study are available from the corresponding author upon reasonable request.

## Ethics approval and patient consent statement

The NCHS Institutional Review Board approved the NHANES protocol. The Biomedical Ethics Committee of Peking University approved the CHARLS protocol (IRB00001052-11015). All NHANES and CHARLS participants provided written informed consent.

## Clinical trial registration

Not applicable.

## Funding statement

Not applicable.

## Conflict of interest disclosure

The authors declare that they have no competing interests.

## References

[1] Ageing and health n.d.. https://www.who.int/news-room/fact-sheets/detail/ageing-and-health.

[2] 2024: the united nations publishes new world population projections | INED n.d. https://www.ined.fr/en/everything_about_population/demographic-facts-sheets/focus-on/2024-les-nations-unies-publient-de-nouvelles-projections-de-population-mondiale.

[3] Chen Q.-F., Ni C., Jiang Y., Chen L., Liao H., Gao J. (2025). Global burden of disease and its risk factors for adults aged 70 and older across 204 countries and territories: a comprehensive analysis of the global burden of disease study 2021. BMC Geriatr.

[4] Franceschi C., Campisi J. (2014). Chronic inflammation (Inflammaging) and its potential contribution to age-associated diseases. J Gerontol, Ser A Biol Sci Med Sci.

[5] Tan S.-H., Wu J.-L., Zhuo S.-X., Zhang Y., Wang M. (2026). Residual risk in atherosclerotic cardiovascular disease after statin therapy: clinical mechanisms and management strategies. World J Cardiol.

[6] Mach F., Baigent C., Catapano A.L., Koskinas K.C., Casula M., Badimon L. (2019). ESC/EAS guidelines for the management of dyslipidaemias: lipid modification to reduce cardiovascular risk. Eur Heart J.

[7] Ravnskov U., Diamond D.M., Hama R., Hamazaki T., Hammarskjöld B., Hynes N. (2016). Lack of an association or an inverse association between low-density-lipoprotein cholesterol and mortality in the elderly: a systematic review. BMJ Open.

[8] Wang B., Liu J., Chen S., Ying M., Chen G., Liu L. (2021). Malnutrition affects cholesterol paradox in coronary artery disease: a 41,229 chinese cohort study. Lipids Health Dis.

[9] Johannesen C.D.L., Mortensen M.B., Langsted A., Nordestgaard B.G. (2021). Apolipoprotein B and non-HDL cholesterol better reflect residual risk than LDL cholesterol in statin-treated patients. J Am Coll Cardiol.

[10] Engell A.E., Bathum L., Siersma V., Andersen C.L., Lind B.S., Jørgensen H.L. (2025). Elevated remnant cholesterol and triglycerides are predictors of increased total mortality in a primary health care population of 327,347 patients. Lipids Health Dis.

[11] Li Y., Zhong X., Cheng G., Zhao C., Zhang L., Hong Y. (2017). Hs-CRP and all-cause, cardiovascular, and cancer mortality risk: a meta-analysis. Atherosclerosis.

[12] Pepys M.B., Hirschfield G.M. (2003). C-reactive protein: a critical update. J Clin Investig.

[13] Doi T., Langsted A., Nordestgaard B.G. (2023). Dual elevated remnant cholesterol and C-reactive protein in myocardial infarction, atherosclerotic cardiovascular disease, and mortality. Atherosclerosis.

[14] Huang X., Li C., Zeng P., Ling Y., Tan S., Bai Z. (2025). Non-traditional lipid-inflammatory parameters estimate the risk of stroke in middle-aged and older Chinese adults: a nationwide prospective cohort study. J Adv Res.

[15] Zhao M., Wang M., Li Z., Wu Y., Lu F., Li Q. (2026). Association of non-conventional lipid-inflammatory parameters with incident hypertension risk: a prospective cohort study. J Glob Health.

[16] Liu Q., Bai B., Liu F., Chen Y., Wang Y., Wang H. (2022). Long-term trends in risk factor management in respondents with chronic kidney disease in the USA. Am J Nephrol.

[17] Zhao Y., Hu Y., Smith J.P., Strauss J., Yang G. (2014). Cohort profile: the China Health and Retirement Longitudinal Study (CHARLS). Int J Epidemiol.

[18] Shao Y., Feng X., Chen Y., Zhang G., Liu Y., Lu H. (2026). Association between the remnant cholesterol inflammatory index and gallstone disease: a cross-sectional analysis based on NHANES 2017 to 2020. Medicine (Baltim).

[19] Xu Y., Zheng X., Li Y., Ye X., Cheng H., Wang H. (2023). Exploring patient medication adherence and data mining methods in clinical big data: a contemporary review. J Evidence-based Med.

[20] Carroll M.D., Rammon J., Storandt R., Tolliver E.A. (2025). National health statistics reports [internet].

[21] Ma L., Sun F., Zhu K., Han Q., Sun Q. (2024). The predictive value of atherogenic index of plasma, Non- high density lipoprotein cholesterol (Non-HDL-C), Non-HDL-C/HDL-C, and lipoprotein combine index for stroke incidence and prognosis in maintenance hemodialysis patients. CIA.

[22] Sheng G., Kuang M., Yang R., Zhong Y., Zhang S., Zou Y. (2022). Evaluation of the value of conventional and unconventional lipid parameters for predicting the risk of diabetes in a non-diabetic population. J Transl Med.

[23] Zheng X., Zhang X., Han Y., Hu H., Cao C. (2023). Nonlinear relationship between atherogenic index of plasma and the risk of prediabetes: a retrospective study based on Chinese adults. Cardiovasc Diabetol.

[24] Nunes S.O.V., Piccoli De Melo L.G., Pizzo De Castro MR., Barbosa D.S., Vargas H.O., Berk M. (2015). Atherogenic index of plasma and atherogenic coefficient are increased in major depression and bipolar disorder, especially when comorbid with tobacco use disorder. J Affect Disord.

[25] Raaj I., Thalamati M., Gowda M.N.V., Rao A. (2024). The role of the atherogenic index of plasma and the Castelli Risk index I and II in cardiovascular disease. Cureus.

[26] Sangouni A.A., Alizadeh M., Jamalzehi A., Hosseinzadeh M., Parastouei K. (2023). Garlic supplementation improves intestinal transit time, lipid accumulation product and cardiometabolic indices in subjects with metabolic syndrome: a randomized controlled trial. Phytother Res.

[27] Stonehouse W., Benassi-Evans B., Louise J. (2025). The effects of a novel nutraceutical combination on low-density lipoprotein cholesterol and other markers of cardiometabolic health in adults with hypercholesterolaemia: a randomised double-blind placebo-controlled trial. Atherosclerosis.

[28] Chen J., Wu Q., Liu H., Hu W., Zhu J., Ji Z. (2024). Predictive value of remnant cholesterol inflammatory index for stroke risk: evidence from the China health and retirement longitudinal study. J Adv Res.

[29] Liu Q., Zhang Y., Chen S., Xiang H., Ouyang J., Liu H. (2024). Association of the triglyceride-glucose index with all-cause and cardiovascular mortality in patients with cardiometabolic syndrome: a national cohort study. Cardiovasc Diabetol.

[30] Ou H., Wei M., Li X., Xia X. (2025). C-reactive protein-triglyceride glucose index in evaluating cardiovascular disease and all-cause mortality incidence among individuals across stages 0–3 of cardiovascular–kidney–metabolic syndrome: a nationwide prospective cohort study. Cardiovasc Diabetol.

[31] You F.-F., Gao J., Gao Y.-N., Li Z.-H., Shen D., Zhong W.-F. (2024). Association between atherogenic index of plasma and all-cause mortality and specific-mortality: a nationwide population-based cohort study. Cardiovasc Diabetol.

[32] Zheng Q., Cao Z., Teng J., Lu Q., Huang P., Zhou J. (2025). Association between atherogenic index of plasma with all-cause and cardiovascular mortality in individuals with cardiovascular-kidney-metabolic syndrome. Cardiovasc Diabetol.

[33] Wang Y., Bi L., Li Q., Wang Q., Lv T., Zhang P. (2025). Remnant cholesterol inflammatory index and its association with all-cause and cause-specific mortality in middle-aged and elderly populations: evidence from US and chinese national population surveys. Lipids Health Dis.

[34] Huo G., Chen Y., Tang Y., Zhou D. (2025). Association between combined atherogenic and frailty index and cardiovascular events: evidence from the CHARLS cohort. Cardiovasc Diabetol.

[35] He D., Wang Z., Li J., Yu K., He Y., He X. (2024). Changes in frailty and incident cardiovascular disease in three prospective cohorts. Eur Heart J.

[36] Liu S., Pan X., Chen B., Zeng D., Xu S., Li R. (2024). Association between healthy lifestyle and frailty in adults and mediating role of weight-adjusted waist index: results from NHANES. BMC Geriatr.

[37] Lu Y., Zhang H., Lu J., Ding Q., Li X., Wang X. (2021). Prevalence of dyslipidemia and availability of lipid-lowering medications among primary health care settings in China. JAMA Netw Open.

[38] Dehghan M., Mente A., Zhang X., Swaminathan S., Li W., Mohan V. (2017). Associations of fats and carbohydrate intake with cardiovascular disease and mortality in 18 countries from five continents (PURE): a prospective cohort study. Lancet.

[39] Lopez-Jaramillo P., Gomez-Arbelaez D., Martinez-Bello D., Abat M.E.M., Alhabib K.F., Avezum Á. (2023). Association of the triglyceride glucose index as a measure of insulin resistance with mortality and cardiovascular disease in populations from five continents (PURE study): a prospective cohort study. Lancet Healthy Longev.

[40] Wu W., Wu D., Cao C., Zhou R., Ding S., Ying Y. (2025). Lipoprotein combine index is associated with non-alcoholic fatty liver disease: a 5-year longitudinal cohort study in non-obese chinese populations with normal lipids. Front Med.

[41] Yilmaz R., Toprak K., Karagoz A., Yontar O.C., Ucar M., Kokcu H.I. (2025). Prognostic value of non-traditional lipid indices for In-hospital mortality in patients with acute coronary syndromes. Medicina (Mex).

[42] Lin H., Xie W., Huang S., Lou J., Zhang R., Yu B. (2025). Association of nontraditional lipid parameters with long‐term prognosis of acute ischemic stroke: a principal components analysis. J Am Heart Assoc: Cardiovasc Cerebrovasc Dis.

[43] Chen Y., Xie K., Han Y., Ju H., Sun J., Zhao X. (2024). The association between triglyceride-glucose index and its combination with systemic inflammation indicators and all-cause and cardiovascular mortality in the general US population: NHANES 1999–2018. Lipids Health Dis.

[44] Nordestgaard B.G. (2016). Triglyceride-rich lipoproteins and atherosclerotic cardiovascular disease: new insights from epidemiology, genetics, and biology. Circ Res.

[45] Ginsberg H.N., Packard C.J., Chapman M.J., Borén J., Aguilar-Salinas C.A., Averna M. (2021). Triglyceride-rich lipoproteins and their remnants: metabolic insights, role in atherosclerotic cardiovascular disease, and emerging therapeutic strategies—a consensus statement from the European atherosclerosis society. Eur Heart J.

[46] Varbo A., Benn M., Tybjærg-Hansen A., Nordestgaard B.G. (2013). Elevated remnant cholesterol causes both low-grade inflammation and ischemic heart disease, whereas elevated low-density lipoprotein cholesterol causes ischemic heart disease without inflammation. Circulation.

[47] Rosenson R.S., Brewer H.B., Ansell B.J., Barter P., Chapman M.J., Heinecke J.W. (2016). Dysfunctional HDL and atherosclerotic cardiovascular disease. Nat Rev Cardiol.

[48] Cao B., Pei J., Kong L., Han Y., Yan G., Wang X. (2026). Molecular interplay between hypertension and dyslipidemia in cardiovascular pathogenesis: toward precision-based therapeutic strategies. Front Cardiovasc Med.

