# Supplementary material for: Associations of non-traditional lipid-inflammatory parameters with all-cause mortality in middle-aged and older adults: Insights from two nationally representative cohorts
Source: Metabol Open. 2026 Oct 9;32:100507. doi: 10.1016/j.metop.2026.100507 (PMC13698077; doi:10.1016/j.metop.2026.100507)
Supplement: Supplementary file 1 [file mmc1.docx]

**Supplementary Materials**

[Supplementary Table. 1 Details of follow-up time distribution in both cohorts.	3](#_Toc241372371)

[Supplementary Table. 2 Description of missing covariates in NHANES.	4](#_Toc241372372)

[Supplementary Table. 3 Description of missing covariates in CHARLS.	5](#_Toc241372373)

[Supplementary Table. 4 Data distribution characteristics of each parameter.	6](#_Toc241372374)

[Supplementary Table. 5 Results based on the proportional hazards assumption of Schoenfeld tests.	7](#_Toc241372375)

[Supplementary Table. 6 Association of non-traditional lipid-inflammatory parameters with all-cause mortality.	8](#_Toc241372376)

[Supplementary Table. 7 Prediction performance of each model at different follow-up time points.	11](#_Toc241372377)

[Supplementary Table. 8 Comparisons of predictive performance using DeLong tests.	12](#_Toc241372378)

[Supplementary Table. 9 Bootstrap-corrected calibration slopes for Cox proportional hazards models.	14](#_Toc241372379)

[Supplementary Table. 10 Subgroup analyses of the non-traditional lipid-inflammatory parameters with all-cause mortality in NHANES.	15](#_Toc241372380)

[Supplementary Table. 11 Subgroup analyses of the non-traditional lipid-inflammatory parameters with all-cause mortality in CHARLS.	20](#_Toc241372381)

[Supplementary Table. 12 Sensitivity analyses of additional control variables specific to the database.	25](#_Toc241372382)

[Supplementary Table. 13 Sensitivity analyses of additional control other possible clinically relevant confounders.	27](#_Toc241372383)

[Supplementary Table. 14 Sensitivity analyses excluding participants who died in the first year.	32](#_Toc241372384)

[Supplementary Table. 15 Sensitivity analyses excluding participants with known baseline use of lipid-lowering medications.	34](#_Toc241372385)

[Supplementary Table. 16 Sensitivity analyses restricted to participants with complete data.	36](#_Toc241372386)

[Supplementary Figure. 1 Analysis of variance inflation factors and Spearman correlation coefficients for the variables included in NHANES.	38](#_Toc241372387)

[Supplementary Figure. 2 Analysis of variance inflation factors and Spearman correlation coefficients for the variables included in CHARLS.	39](#_Toc241372388)

[Supplementary Figure. 3 Event distribution during the follow-up period.	40](#_Toc241372389)

[Supplementary Figure. 4 Weighted Kaplan-Meier survival curves by quartiles of non-traditional lipid-inflammatory parameters in NHANES.	41](#_Toc241372390)

[Supplementary Figure. 5 Weighted Kaplan-Meier survival curves by quartiles of non-traditional lipid-inflammatory parameters in CHARLS.	42](#_Toc241372391)

[Supplementary Figure. 6 Weighted RCS analyses of the non-traditional lipid-inflammatory parameters with all-cause mortality in CHARLS.	43](#_Toc241372392)

[Supplementary Figure. 7 Calibration curves for Cox proportional hazards models to predict all-cause mortality at different follow-up time points.	44](#_Toc241372393)

# **Supplementary Table. 1** Details of follow-up time distribution in both cohorts.

| **Items** | **NHANES** | **CHARLS** |
| --- | --- | --- |
| Sample, n | 9,943 | 9,409 |
| Death, n (%) | 2,377 (23.9%) | 1,246 (13.2%) |
| Alive/Censored, n (%) | 7,566 (76.1%) | 8,163 (86.8%) |
| Weighted mean follow-up time, month (SD) | 80.3 (53.9) | 100.2 (19.8) |
| Weighted median follow-up time, month (IQR) | 56.0 (33.0, 130.0) | 108.0 (106.0, 108.0) |
| Min follow-up time, month | 1.0 | 1.0 |
| Max follow-up time, month | 180.0 | 113.0 |

Abbreviations: NHANES, National Health and Nutrition Examination Survey; CHARLS, China Health and Retirement Longitudinal Study.

# **Supplementary Table. 2** Description of missing covariates in NHANES.

| **Variables** | **Total count** | **Missing** | **Missing rate(%)** |
| --- | --- | --- | --- |
| Age | 9943 | 0 | 0.00% |
| Sex | 9943 | 0 | 0.00% |
| Race | 9943 | 0 | 0.00% |
| Marital status | 9943 | 6 | 0.06% |
| Education | 9943 | 15 | 0.15% |
| PIR | 9943 | 1018 | 10.23% |
| Smoking | 9943 | 10 | 0.10% |
| Drinking | 9943 | 509 | 5.11% |
| Diabetes | 9943 | 7 | 0.07% |
| Hypertension | 9943 | 15 | 0.15% |
| Heart disease | 9943 | 25 | 0.25% |
| Stroke | 9943 | 23 | 0.23% |
| Cancer | 9943 | 11 | 0.11% |
| Lipid medication | 9943 | 5635 | 56.67% |
| BMI | 9943 | 189 | 1.90% |
| SBP | 9943 | 300 | 3.01% |

Abbreviations: NHANES, National Health and Nutrition Examination Survey; PIR, poverty income ratio; BMI, body mass index; SBP, systolic blood pressure.

# **Supplementary Table. 3** Description of missing covariates in CHARLS.

| **Variables** | **Total count** | **Missing** | **Missing rate(%)** |
| --- | --- | --- | --- |
| Age | 9409 | 0 | 0.00% |
| Sex | 9409 | 0 | 0.00% |
| Residence | 9409 | 0 | 0.00% |
| Marital status | 9409 | 0 | 0.00% |
| Education | 9409 | 7 | 0.07% |
| Smoking | 9409 | 26 | 0.27% |
| Drinking | 9409 | 4 | 0.04% |
| Diabetes | 9409 | 63 | 0.66% |
| Hypertension | 9409 | 25 | 0.26% |
| Heart disease | 9409 | 41 | 0.43% |
| Stroke | 9409 | 22 | 0.23% |
| Cancer | 9409 | 30 | 0.31% |
| Lipid medication | 9409 | 8890 | 94.48% |
| BMI | 9409 | 159 | 1.68% |
| SBP | 9409 | 122 | 1.29% |

Abbreviations: CHARLS, China Health and Retirement Longitudinal Study; BMI, body mass index; SBP, systolic blood pressure.

# **Supplementary Table. 4** Data distribution characteristics of each parameter.

| **Parameters** | **Mean value** | **Median value** | **1-SD value** |
| --- | --- | --- | --- |
| **NHANES** | | | |
| non-HDL-C-CRP | 66.653 | 31.5 | 99.252 |
| RC-CRP | 13.815 | 6.091 | 21.088 |
| AIP-CRP | 0.196 | 0.075 | 0.339 |
| AC-CRP | 1.394 | 0.603 | 2.194 |
| CRI-I-CRP | 1.875 | 0.852 | 2.88 |
| CRI-II-CRP | 1.08 | 0.469 | 1.689 |
| LCI-CRP | 34996.318 | 11606.198 | 63516.455 |
| RC/HDL-C-CRP | 0.305 | 0.119 | 0.511 |
| **CHARLS** | | | |
| non-HDL-C-CRP | 36.012 | 14.648 | 70.601 |
| RC-CRP | 6.591 | 2.111 | 13.727 |
| AIP-CRP | 0.093 | 0.03 | 0.203 |
| AC-CRP | 0.819 | 0.299 | 1.671 |
| CRI-I-CRP | 1.077 | 0.409 | 2.175 |
| CRI-II-CRP | 0.642 | 0.241 | 1.314 |
| LCI-CRP | 17924.037 | 5288.055 | 38262.391 |
| RC/HDL-C-CRP | 0.167 | 0.043 | 0.384 |

Abbreviations: NHANES, National Health and Nutrition Examination Survey; CHARLS, China Health and Retirement Longitudinal Study.

# **Supplementary Table. 5** Results based on the proportional hazards assumption of Schoenfeld tests.

| **Parameters** | **Exposure form** | **Exposure-*P*** | **Global-*P*** |
| --- | --- | --- | --- |
| **NHANES** | | | |
| non-HDL-C-CRP | Per 1-SD | <0.001 | 0.020 |
|  | Quartiles | 0.051 | 0.091 |
| RC-CRP | Per 1-SD | 0.010 | 0.067 |
|  | Quartiles | 0.357 | 0.261 |
| AIP-CRP | Per 1-SD | 0.018 | 0.092 |
|  | Quartiles | 0.462 | 0.298 |
| AC-CRP | Per 1-SD | 0.001 | 0.035 |
|  | Quartiles | 0.044 | 0.094 |
| CRI-I-CRP | Per 1-SD | <0.001 | 0.024 |
|  | Quartiles | 0.014 | 0.045 |
| CRI-II-CRP | Per 1-SD | <0.001 | 0.026 |
|  | Quartiles | 0.050 | 0.098 |
| LCI-CRP | Per 1-SD | 0.018 | 0.124 |
|  | Quartiles | 0.072 | 0.118 |
| RC/HDL-C-CRP | Per 1-SD | 0.023 | 0.112 |
|  | Quartiles | 0.289 | 0.241 |
| **CHARLS** | | | |
| non-HDL-C-CRP | Per 1-SD | 0.047 | <0.001 |
|  | Quartiles | 0.859 | <0.001 |
| RC-CRP | Per 1-SD | 0.068 | <0.001 |
|  | Quartiles | 0.286 | <0.001 |
| AIP-CRP | Per 1-SD | 0.033 | <0.001 |
|  | Quartiles | 0.003 | <0.001 |
| AC-CRP | Per 1-SD | 0.041 | <0.001 |
|  | Quartiles | 0.532 | <0.001 |
| CRI-I-CRP | Per 1-SD | 0.032 | <0.001 |
|  | Quartiles | 0.728 | <0.001 |
| CRI-II-CRP | Per 1-SD | 0.039 | <0.001 |
|  | Quartiles | 0.958 | <0.001 |
| LCI-CRP | Per 1-SD | 0.142 | <0.001 |
|  | Quartiles | 0.691 | <0.001 |
| RC/HDL-C-CRP | Per 1-SD | 0.057 | <0.001 |
|  | Quartiles | 0.344 | <0.001 |

Abbreviations: NHANES, National Health and Nutrition Examination Survey; CHARLS, China Health and Retirement Longitudinal Study.

# **Supplementary Table. 6** Association of non-traditional lipid-inflammatory parameters with all-cause mortality.

| **Parameters** | **NHANES** | | | | **CHARLS** | | | |
| --- | --- | --- | --- | --- | --- | --- | --- | --- |
|  | **Model 1** | | **Model 2** | | **Model 1** | | **Model 2** | |
|  | **HR (95% CI)** | ***P*** | **HR (95% CI)** | ***P*** | **HR (95% CI)** | ***P*** | **HR (95% CI)** | ***P*** |
| **non-HDL-C-CRP** |  |  |  |  |  |  |  |  |
| Per 1-SD | 1.24 (1.17-1.31) | <0.001 | 1.21 (1.15-1.28) | <0.001 | 1.18 (1.13-1.24) | <0.001 | 1.18 (1.12-1.24) | <0.001 |
| Q1 | Ref |  | Ref |  | Ref |  | Ref |  |
| Q2 | 1.14 (1.00-1.31) | 0.056 | 1.08 (0.94-1.24) | 0.264 | 1.06 (0.84-1.35) | 0.612 | 1.05 (0.83-1.32) | 0.674 |
| Q3 | 1.30 (1.10-1.53) | 0.002 | 1.16 (0.99-1.36) | 0.068 | 1.44 (1.12-1.84) | 0.005 | 1.46 (1.14-1.88) | 0.003 |
| Q4 | 1.69 (1.47-1.94) | <0.001 | 1.47 (1.28-1.69) | <0.001 | 1.63 (1.30-2.03) | <0.001 | 1.64 (1.33-2.01) | <0.001 |
| ***P_trend_*** | <0.001 |  | <0.001 |  | <0.001 |  | <0.001 |  |
| **RC-CRP** |  |  |  |  |  |  |  |  |
| Per 1-SD | 1.23 (1.16-1.31) | <0.001 | 1.21 (1.15-1.28) | <0.001 | 1.19 (1.13-1.26) | <0.001 | 1.18 (1.11-1.25) | <0.001 |
| Q1 | Ref |  | Ref |  | Ref |  | Ref |  |
| Q2 | 1.14 (0.96-1.35) | 0.136 | 1.05 (0.88-1.25) | 0.597 | 1.16 (0.93-1.45) | 0.198 | 1.12 (0.90-1.41) | 0.307 |
| Q3 | 1.28 (1.12-1.46) | <0.001 | 1.12 (0.98-1.28) | 0.106 | 1.46 (1.16-1.83) | 0.001 | 1.48 (1.18-1.85) | <0.001 |
| Q4 | 1.65 (1.44-1.90) | <0.001 | 1.44 (1.25-1.66) | <0.001 | 1.70 (1.35-2.13) | <0.001 | 1.68 (1.36-2.08) | <0.001 |
| ***P_trend_*** | <0.001 |  | <0.001 |  | <0.001 |  | <0.001 |  |
| **AIP-CRP** |  |  |  |  |  |  |  |  |
| Per 1-SD | 1.25 (1.17-1.33) | <0.001 | 1.22 (1.15-1.29) | <0.001 | 1.15 (1.09-1.22) | <0.001 | 1.15 (1.09-1.21) | <0.001 |
| Q1 | Ref |  | Ref |  | Ref |  | Ref |  |
| Q2 | 0.89 (0.77-1.03) | 0.106 | 0.85 (0.74-0.97) | 0.017 | 0.90 (0.72-1.13) | 0.376 | 0.91 (0.73-1.13) | 0.390 |
| Q3 | 1.02 (0.91-1.15) | 0.694 | 0.92 (0.82-1.04) | 0.172 | 0.99 (0.79-1.24) | 0.937 | 1.01 (0.81-1.27) | 0.916 |
| Q4 | 1.42 (1.23-1.64) | <0.001 | 1.23 (1.08-1.41) | 0.002 | 1.22 (0.96-1.54) | 0.104 | 1.27 (1.02-1.59) | 0.035 |
| ***P_trend_*** | <0.001 |  | <0.001 |  | 0.054 |  | 0.014 |  |
| **AC-CRP** |  |  |  |  |  |  |  |  |
| Per 1-SD | 1.26 (1.19-1.34) | <0.001 | 1.23 (1.17-1.30) | <0.001 | 1.16 (1.10-1.22) | <0.001 | 1.16 (1.10-1.22) | <0.001 |
| Q1 | Ref |  | Ref |  | Ref |  | Ref |  |
| Q2 | 1.09 (0.96-1.23) | 0.172 | 1.03 (0.91-1.17) | 0.659 | 1.09 (0.86-1.37) | 0.465 | 1.09 (0.87-1.38) | 0.450 |
| Q3 | 1.30 (1.10-1.53) | 0.002 | 1.16 (0.99-1.37) | 0.064 | 1.28 (0.99-1.65) | 0.064 | 1.30 (1.01-1.69) | 0.044 |
| Q4 | 1.67 (1.47-1.90) | <0.001 | 1.43 (1.26-1.62) | <0.001 | 1.55 (1.25-1.93) | <0.001 | 1.58 (1.29-1.94) | <0.001 |
| ***P_trend_*** | <0.001 |  | <0.001 |  | <0.001 |  | <0.001 |  |
| **CRI-I-CRP** |  |  |  |  |  |  |  |  |
| Per 1-SD | 1.27 (1.20-1.34) | <0.001 | 1.24 (1.18-1.31) | <0.001 | 1.17 (1.12-1.24) | <0.001 | 1.17 (1.11-1.23) | <0.001 |
| Q1 | Ref |  | Ref |  | Ref |  | Ref |  |
| Q2 | 1.17 (1.03-1.33) | 0.020 | 1.11 (0.97-1.27) | 0.145 | 1.15 (0.92-1.43) | 0.220 | 1.15 (0.92-1.43) | 0.210 |
| Q3 | 1.32 (1.12-1.56) | 0.001 | 1.18 (0.99-1.39) | 0.061 | 1.33 (1.03-1.72) | 0.031 | 1.37 (1.06-1.78) | 0.016 |
| Q4 | 1.78 (1.56-2.03) | <0.001 | 1.53 (1.34-1.75) | <0.001 | 1.67 (1.35-2.06) | <0.001 | 1.70 (1.39-2.08) | <0.001 |
| ***P_trend_*** | <0.001 |  | <0.001 |  | <0.001 |  | <0.001 |  |
| **CRI-II-CRP** |  |  |  |  |  |  |  |  |
| Per 1-SD | 1.26 (1.19-1.33) | <0.001 | 1.22 (1.16-1.29) | <0.001 | 1.16 (1.10-1.22) | <0.001 | 1.15 (1.09-1.22) | <0.001 |
| Q1 | Ref |  | Ref |  | Ref |  | Ref |  |
| Q2 | 1.15 (1.01-1.30) | 0.031 | 1.09 (0.96-1.23) | 0.183 | 1.04 (0.82-1.32) | 0.755 | 1.06 (0.84-1.34) | 0.640 |
| Q3 | 1.26 (1.06-1.49) | 0.008 | 1.14 (0.97-1.34) | 0.119 | 1.34 (1.04-1.73) | 0.022 | 1.36 (1.05-1.76) | 0.019 |
| Q4 | 1.69 (1.49-1.92) | <0.001 | 1.45 (1.27-1.65) | <0.001 | 1.50 (1.21-1.85) | <0.001 | 1.53 (1.25-1.87) | <0.001 |
| ***P_trend_*** | <0.001 |  | <0.001 |  | <0.001 |  | <0.001 |  |
| **LCI-CRP** |  |  |  |  |  |  |  |  |
| Per 1-SD | 1.20 (1.12-1.28) | <0.001 | 1.16 (1.09-1.24) | <0.001 | 1.15 (1.09-1.21) | <0.001 | 1.15 (1.09-1.21) | <0.001 |
| Q1 | Ref |  | Ref |  | Ref |  | Ref |  |
| Q2 | 1.05 (0.93-1.19) | 0.439 | 1.00 (0.88-1.13) | 0.990 | 0.88 (0.69-1.11) | 0.276 | 0.90 (0.71-1.13) | 0.349 |
| Q3 | 1.15 (1.01-1.30) | 0.035 | 1.05 (0.92-1.19) | 0.475 | 1.06 (0.82-1.37) | 0.661 | 1.08 (0.83-1.40) | 0.562 |
| Q4 | 1.34 (1.18-1.53) | <0.001 | 1.18 (1.04-1.34) | 0.010 | 1.32 (1.06-1.64) | 0.012 | 1.39 (1.13-1.71) | 0.002 |
| ***P_trend_*** | <0.001 |  | 0.005 |  | 0.002 |  | <0.001 |  |
| **RC/HDL-C-CRP** |  |  |  |  |  |  |  |  |
| Per 1-SD | 1.25 (1.17-1.32) | <0.001 | 1.22 (1.15-1.29) | <0.001 | 1.17 (1.10-1.24) | <0.001 | 1.16 (1.10-1.23) | <0.001 |
| Q1 | Ref |  | Ref |  | Ref |  | Ref |  |
| Q2 | 1.05 (0.91-1.22) | 0.492 | 0.99 (0.86-1.14) | 0.900 | 1.17 (0.93-1.48) | 0.175 | 1.17 (0.93-1.46) | 0.184 |
| Q3 | 1.19 (1.04-1.36) | 0.010 | 1.07 (0.95-1.21) | 0.281 | 1.34 (1.04-1.73) | 0.023 | 1.36 (1.06-1.75) | 0.017 |
| Q4 | 1.58 (1.38-1.82) | <0.001 | 1.37 (1.19-1.57) | <0.001 | 1.58 (1.27-1.96) | <0.001 | 1.60 (1.30-1.96) | <0.001 |
| ***P_trend_*** | <0.001 |  | <0.001 |  | <0.001 |  | <0.001 |  |

Note: Model 1 was adjusted for age and sex; Model 2 was adjusted for age, sex, marital status, education, smoking and drinking status.

Abbreviations: NHANES, National Health and Nutrition Examination Survey; CHARLS, China Health and Retirement Longitudinal Study; HR, hazard ratio; CI, confidence interval; Ref, reference.

# **Supplementary Table. 7** Prediction performance of each model at different follow-up time points.

| **Model** | **Follow-up time points** | | | |
| --- | --- | --- | --- | --- |
|  | **1 year** | **3 year** | **5 year** | **7 year** |
|  | **AUC (95% CI)** | **AUC (95% CI)** | **AUC (95% CI)** | **AUC (95% CI)** |
| **NHANES** | | | | |
| Base model | 0.716 (0.680-0.751) | 0.749 (0.730-0.770) | 0.760 (0.743-0.777) | 0.769 (0.754-0.783) |
| non-HDL-C-CRP | 0.742 (0.705-0.777) | 0.762 (0.743-0.782) | 0.766 (0.751-0.783) | 0.774 (0.759-0.787) |
| RC-CRP | 0.740 (0.703-0.774) | 0.761 (0.742-0.781) | 0.765 (0.749-0.781) | 0.773 (0.758-0.787) |
| AIP-CRP | 0.739 (0.703-0.774) | 0.761 (0.742-0.780) | 0.764 (0.748-0.781) | 0.773 (0.758-0.787) |
| AC-CRP | 0.744 (0.708-0.779) | 0.763 (0.744-0.783) | 0.766 (0.751-0.783) | 0.774 (0.760-0.788) |
| CRI-I-CRP | 0.746 (0.710-0.781) | 0.764 (0.745-0.784) | 0.767 (0.752-0.784) | 0.775 (0.760-0.788) |
| CRI-II-CRP | 0.743 (0.707-0.779) | 0.763 (0.743-0.782) | 0.767 (0.751-0.783) | 0.774 (0.760-0.788) |
| LCI-CRP | 0.731 (0.695-0.767) | 0.756 (0.737-0.777) | 0.762 (0.746-0.778) | 0.771 (0.756-0.784) |
| RC/HDL-C-CRP | 0.740 (0.704-0.774) | 0.761 (0.742-0.781) | 0.765 (0.749-0.781) | 0.773 (0.759-0.787) |
| **CHARLS** | | | | |
| Base model | 0.765 (0.706-0.827) | 0.784 (0.746-0.819) | 0.762 (0.739-0.788) | 0.784 (0.763-0.807) |
| non-HDL-C-CRP | 0.790 (0.725-0.852) | 0.805 (0.767-0.840) | 0.775 (0.751-0.800) | 0.793 (0.772-0.815) |
| RC-CRP | 0.789 (0.723-0.851) | 0.802 (0.763-0.837) | 0.775 (0.750-0.800) | 0.793 (0.773-0.815) |
| AIP-CRP | 0.784 (0.720-0.847) | 0.801 (0.763-0.836) | 0.773 (0.749-0.798) | 0.792 (0.771-0.814) |
| AC-CRP | 0.788 (0.723-0.850) | 0.804 (0.767-0.838) | 0.774 (0.750-0.799) | 0.792 (0.772-0.814) |
| CRI-I-CRP | 0.789 (0.724-0.852) | 0.805 (0.768-0.840) | 0.774 (0.750-0.799) | 0.793 (0.772-0.815) |
| CRI-II-CRP | 0.788 (0.724-0.851) | 0.805 (0.768-0.839) | 0.773 (0.750-0.799) | 0.792 (0.771-0.814) |
| LCI-CRP | 0.785 (0.720-0.848) | 0.797 (0.759-0.833) | 0.771 (0.747-0.797) | 0.790 (0.770-0.813) |
| RC/HDL-C-CRP | 0.786 (0.720-0.848) | 0.800 (0.761-0.834) | 0.773 (0.749-0.798) | 0.792 (0.772-0.815) |

Note: The prediction performance of each model was evaluated using the Bootstrap method. Base model included age, sex, marital status, education, smoking, drinking, diabetes, hypertension, BMI, and SBP.

Abbreviations: NHANES, National Health and Nutrition Examination Survey; CHARLS, China Health and Retirement Longitudinal Study; AUC, the area under the curve; CI, confidence interval.

# **Supplementary Table. 8** Comparisons of predictive performance using DeLong tests.

| **Model** | **Follow-up time points** | | | | | | | |
| --- | --- | --- | --- | --- | --- | --- | --- | --- |
|  | **1 year** | | **3 year** | | **5 year** | | **7 year** | |
|  | **ΔAUC (95% CI)** | ***P*** | **ΔAUC (95% CI)** | ***P*** | **ΔAUC (95% CI)** | ***P*** | **ΔAUC (95% CI)** | ***P*** |
| **NHANES** | | | | | | | | |
| non-HDL-C-CRP | 0.0258 (0.0128, 0.0414) | <0.001 | 0.0125 (0.0069, 0.0187) | <0.001 | 0.0064 (0.0029, 0.0105) | <0.001 | 0.0044 (0.0017, 0.0075) | 0.003 |
| RC-CRP | 0.0233 (0.0115, 0.0378) | <0.001 | 0.0118 (0.0066, 0.0182) | <0.001 | 0.0049 (0.0019, 0.0085) | 0.003 | 0.0038 (0.0013, 0.0069) | 0.007 |
| AIP-CRP | 0.0225 (0.0108, 0.0368) | <0.001 | 0.0113 (0.0059, 0.0179) | <0.001 | 0.0044 (0.0013, 0.0081) | 0.011 | 0.0038 (0.0014, 0.0070) | 0.007 |
| AC-CRP | 0.0275 (0.0138, 0.0445) | <0.001 | 0.0136 (0.0076, 0.0206) | <0.001 | 0.0066 (0.0029, 0.0110) | <0.001 | 0.0050 (0.0019, 0.0083) | 0.002 |
| CRI-I-CRP | 0.0298 (0.0155, 0.0472) | <0.001 | 0.0148 (0.0084, 0.0220) | <0.001 | 0.0075 (0.0036, 0.0119) | <0.001 | 0.0055 (0.0024, 0.0087) | <0.001 |
| CRI-II-CRP | 0.0272 (0.0134, 0.0443) | <0.001 | 0.0134 (0.0074, 0.0205) | <0.001 | 0.0067 (0.0029, 0.0111) | <0.001 | 0.0049 (0.0018, 0.0083) | 0.002 |
| LCI-CRP | 0.0151 (0.0054, 0.0267) | 0.002 | 0.0071 (0.0030, 0.0121) | <0.001 | 0.0018 (-0.0005, 0.0045) | 0.208 | 0.0016 (-0.0002, 0.0042) | 0.180 |
| RC/HDL-C-CRP | 0.0234 (0.0116, 0.0380) | <0.001 | 0.0116 (0.0063, 0.0183) | <0.001 | 0.0050 (0.0018, 0.0088) | 0.004 | 0.0041 (0.0016, 0.0072) | 0.004 |
| **CHARLS** | | | | | | | | |
| non-HDL-C-CRP | 0.0244 (0.0048, 0.0502) | 0.029 | 0.0216 (0.0093, 0.0368) | <0.001 | 0.0125 (0.0064, 0.0197) | <0.001 | 0.0091 (0.0044, 0.0147) | <0.001 |
| RC-CRP | 0.0233 (0.0035, 0.0484) | 0.032 | 0.0186 (0.0068, 0.0329) | 0.002 | 0.0126 (0.0065, 0.0200) | <0.001 | 0.0096 (0.0045, 0.0153) | <0.001 |
| AIP-CRP | 0.0189 (0.0004, 0.0428) | 0.066 | 0.0178 (0.0066, 0.0326) | 0.002 | 0.0110 (0.0054, 0.0179) | <0.001 | 0.0085 (0.0039, 0.0137) | <0.001 |
| AC-CRP | 0.0224 (0.0039, 0.0473) | 0.034 | 0.0207 (0.0083, 0.0363) | <0.001 | 0.0116 (0.0059, 0.0185) | <0.001 | 0.0086 (0.0042, 0.0136) | <0.001 |
| CRI-I-CRP | 0.0235 (0.0044, 0.0492) | 0.032 | 0.0218 (0.0092, 0.0374) | <0.001 | 0.0123 (0.0065, 0.0195) | <0.001 | 0.0092 (0.0043, 0.0146) | <0.001 |
| CRI-II-CRP | 0.0231 (0.0046, 0.0489) | 0.03 | 0.0214 (0.0089, 0.0373) | <0.001 | 0.0113 (0.0057, 0.0184) | <0.001 | 0.0083 (0.0039, 0.0135) | <0.001 |
| LCI-CRP | 0.0199 (0.0024, 0.0441) | 0.044 | 0.0138 (0.0040, 0.0267) | 0.004 | 0.0092 (0.0041, 0.0152) | <0.001 | 0.0069 (0.0028, 0.0114) | <0.001 |
| RC/HDL-C-CRP | 0.0205 (0.0015, 0.0442) | 0.046 | 0.0162 (0.0049, 0.0300) | 0.004 | 0.0113 (0.0056, 0.0183) | <0.001 | 0.0089 (0.0040, 0.0145) | <0.001 |

Note: The bootstrap method was used to obtain the difference in AUC (ΔAUC) and its 95% CI between each parameter model and the base model. DeLong tests were used to evaluate the statistical significance. Base model included age, sex, marital status, education, smoking, drinking, diabetes, hypertension, BMI, and SBP.

Abbreviations: NHANES, National Health and Nutrition Examination Survey; CHARLS, China Health and Retirement Longitudinal Study; AUC, the area under the curve; CI, confidence interval.

# **Supplementary Table. 9** Bootstrap-corrected calibration slopes for Cox proportional hazards models.

| **Model** | **NHANES** | **CHARLS** |
| --- | --- | --- |
|  | **Calibration slope (95% CI)** | **Calibration slope (95% CI)** |
| Base model | 0.9910 (0.9394, 1.0433) | 0.9833 (0.9297, 1.0419) |
| non-HDL-C-CRP | 0.9902 (0.9392, 1.0438) | 0.9817 (0.9291, 1.0422) |
| RC-CRP | 0.9901 (0.9398, 1.0435) | 0.9814 (0.9262, 1.0381) |
| AIP-CRP | 0.9901 (0.9393, 1.0419) | 0.9816 (0.9248, 1.0388) |
| AC-CRP | 0.9902 (0.9390, 1.0439) | 0.9814 (0.9276, 1.0409) |
| CRI-I-CRP | 0.9901 (0.9390, 1.0431) | 0.9815 (0.9278, 1.0416) |
| CRI-II-CRP | 0.9903 (0.9411, 1.0442) | 0.9815 (0.9267, 1.0404) |
| LCI-CRP | 0.9904 (0.9405, 1.0460) | 0.9815 (0.9259, 1.0410) |
| RC/HDL-C-CRP | 0.9901 (0.9391, 1.0429) | 0.9817 (0.9282, 1.0395) |

Note: Calibration slopes were identical across follow-up time points within each model, as the slope was determined by the linear predictor. Base model included age, sex, marital status, education, smoking, drinking, diabetes, hypertension, BMI, and SBP.

Abbreviations: NHANES, National Health and Nutrition Examination Survey; CHARLS, China Health and Retirement Longitudinal Study; CI, confidence interval.

# **Supplementary Table. 10** Subgroup analyses of the non-traditional lipid-inflammatory parameters with all-cause mortality in NHANES.

| **Subgroups** | **Events/Total** | **HR (95% CI)** | ***P*** | ***P* for interaction** | ***P* for interaction**  **(BH-FDR)** |
| --- | --- | --- | --- | --- | --- |
| **non-HDL-C-CRP** | | | | | |
| **Age** |  |  |  | 0.492 | 0.538 |
| 45-60 y | 243/2188 | 1.00 (1.00-1.00) | 0.002 |  |  |
| ≥60 y | 2134/7755 | 1.00 (1.00-1.00) | <0.001 |  |  |
| **Sex** |  |  |  | 0.068 | 0.119 |
| Male | 1390/4926 | 1.00 (1.00-1.00) | <0.001 |  |  |
| Female | 987/5017 | 1.00 (1.00-1.00) | <0.001 |  |  |
| **Smoking** |  |  |  | 0.055 | 0.119 |
| Never | 909/4684 | 1.00 (1.00-1.00) | 0.021 |  |  |
| Former | 1031/3592 | 1.00 (1.00-1.00) | <0.001 |  |  |
| Current | 437/1667 | 1.00 (1.00-1.00) | <0.001 |  |  |
| **Drinking** |  |  |  | 0.538 | 0.538 |
| Never | 681/2923 | 1.00 (1.00-1.00) | <0.001 |  |  |
| Former | 661/1954 | 1.00 (1.00-1.00) | <0.001 |  |  |
| Current | 1035/5066 | 1.00 (1.00-1.00) | <0.001 |  |  |
| **Diabetes** |  |  |  | 0.036 | 0.119 |
| No | 1752/7627 | 1.00 (1.00-1.00) | <0.001 |  |  |
| Yes | 625/2316 | 1.00 (1.00-1.00) | <0.001 |  |  |
| **Hypertension** |  |  |  | 0.057 | 0.119 |
| No | 853/4099 | 1.00 (1.00-1.00) | <0.001 |  |  |
| Yes | 1524/5844 | 1.00 (1.00-1.00) | <0.001 |  |  |
| **Frailty status** |  |  |  | 0.526 | 0.538 |
| Non-frail | 314/2022 | 1.00 (1.00-1.00) | 0.003 |  |  |
| Pre-frail | 842/4087 | 1.00 (1.00-1.00) | 0.024 |  |  |
| Frail | 1221/3834 | 1.00 (1.00-1.00) | <0.001 |  |  |
| **RC-CRP** | | | | | |
| **Age** |  |  |  | 0.381 | 0.381 |
| 45-60 y | 243/2188 | 1.01 (1.01-1.01) | 0.002 |  |  |
| ≥60 y | 2134/7755 | 1.01 (1.01-1.01) | <0.001 |  |  |
| **Sex** |  |  |  | 0.097 | 0.34 |
| Male | 1390/4926 | 1.01 (1.01-1.01) | <0.001 |  |  |
| Female | 987/5017 | 1.01 (1.00-1.01) | <0.001 |  |  |
| **Smoking** |  |  |  | 0.054 | 0.34 |
| Never | 909/4684 | 1.00 (1.00-1.01) | 0.088 |  |  |
| Former | 1031/3592 | 1.01 (1.01-1.01) | <0.001 |  |  |
| Current | 437/1667 | 1.01 (1.01-1.02) | <0.001 |  |  |
| **Drinking** |  |  |  | 0.356 | 0.381 |
| Never | 681/2923 | 1.01 (1.00-1.01) | <0.001 |  |  |
| Former | 661/1954 | 1.01 (1.01-1.01) | <0.001 |  |  |
| Current | 1035/5066 | 1.01 (1.01-1.01) | <0.001 |  |  |
| **Diabetes** |  |  |  | 0.262 | 0.367 |
| No | 1752/7627 | 1.01 (1.01-1.01) | <0.001 |  |  |
| Yes | 625/2316 | 1.01 (1.01-1.01) | <0.001 |  |  |
| **Hypertension** |  |  |  | 0.247 | 0.367 |
| No | 853/4099 | 1.01 (1.00-1.01) | <0.001 |  |  |
| Yes | 1524/5844 | 1.01 (1.01-1.01) | <0.001 |  |  |
| **Frailty status** |  |  |  | 0.201 | 0.367 |
| Non-frail | 314/2022 | 1.01 (1.00-1.02) | 0.002 |  |  |
| Pre-frail | 842/4087 | 1.00 (1.00-1.01) | 0.043 |  |  |
| Frail | 1221/3834 | 1.01 (1.00-1.01) | <0.001 |  |  |
| **AIP-CRP** | | | | | |
| **Age** |  |  |  | 0.101 | 0.365 |
| 45-60 y | 243/2188 | 2.04 (1.56-2.67) | <0.001 |  |  |
| ≥60 y | 2134/7755 | 1.52 (1.34-1.73) | <0.001 |  |  |
| **Sex** |  |  |  | 0.318 | 0.365 |
| Male | 1390/4926 | 1.83 (1.59-2.12) | <0.001 |  |  |
| Female | 987/5017 | 1.68 (1.39-2.03) | <0.001 |  |  |
| **Smoking** |  |  |  | 0.227 | 0.365 |
| Never | 909/4684 | 1.35 (1.08-1.70) | 0.074 |  |  |
| Former | 1031/3592 | 1.98 (1.68-2.34) | <0.001 |  |  |
| Current | 437/1667 | 1.90 (1.52-2.38) | <0.001 |  |  |
| **Drinking** |  |  |  | 0.290 | 0.365 |
| Never | 681/2923 | 1.55 (1.28-1.88) | <0.001 |  |  |
| Former | 661/1954 | 1.75 (1.41-2.18) | <0.001 |  |  |
| Current | 1035/5066 | 1.97 (1.63-2.38) | <0.001 |  |  |
| **Diabetes** |  |  |  | 0.253 | 0.365 |
| No | 1752/7627 | 1.71 (1.48-1.98) | <0.001 |  |  |
| Yes | 625/2316 | 1.83 (1.51-2.21) | <0.001 |  |  |
| **Hypertension** |  |  |  | 0.299 | 0.365 |
| No | 853/4099 | 1.63 (1.30-2.06) | <0.001 |  |  |
| Yes | 1524/5844 | 1.81 (1.59-2.07) | <0.001 |  |  |
| **Frailty status** |  |  |  | 0.365 | 0.365 |
| Non-frail | 314/2022 | 2.12 (1.27-3.56) | 0.002 |  |  |
| Pre-frail | 842/4087 | 1.43 (1.12-1.83) | 0.012 |  |  |
| Frail | 1221/3834 | 1.46 (1.28-1.68) | <0.001 |  |  |
| **AC-CRP** | | | | | |
| **Age** |  |  |  | 0.281 | 0.456 |
| 45-60 y | 243/2188 | 1.11 (1.06-1.15) | <0.001 |  |  |
| ≥60 y | 2134/7755 | 1.07 (1.05-1.10) | <0.001 |  |  |
| **Sex** |  |  |  | 0.326 | 0.456 |
| Male | 1390/4926 | 1.11 (1.08-1.13) | <0.001 |  |  |
| Female | 987/5017 | 1.09 (1.06-1.12) | <0.001 |  |  |
| **Smoking** |  |  |  | 0.242 | 0.456 |
| Never | 909/4684 | 1.07 (1.03-1.10) | 0.009 |  |  |
| Former | 1031/3592 | 1.12 (1.09-1.15) | <0.001 |  |  |
| Current | 437/1667 | 1.11 (1.07-1.15) | <0.001 |  |  |
| **Drinking** |  |  |  | 0.500 | 0.5 |
| Never | 681/2923 | 1.09 (1.06-1.12) | <0.001 |  |  |
| Former | 661/1954 | 1.09 (1.06-1.13) | <0.001 |  |  |
| Current | 1035/5066 | 1.11 (1.08-1.14) | <0.001 |  |  |
| **Diabetes** |  |  |  | 0.093 | 0.327 |
| No | 1752/7627 | 1.09 (1.07-1.12) | <0.001 |  |  |
| Yes | 625/2316 | 1.12 (1.08-1.15) | <0.001 |  |  |
| **Hypertension** |  |  |  | 0.054 | 0.327 |
| No | 853/4099 | 1.07 (1.04-1.11) | <0.001 |  |  |
| Yes | 1524/5844 | 1.11 (1.09-1.14) | <0.001 |  |  |
| **Frailty status** |  |  |  | 0.489 | 0.5 |
| Non-frail | 314/2022 | 1.12 (1.05-1.19) | 0.001 |  |  |
| Pre-frail | 842/4087 | 1.06 (1.02-1.10) | 0.012 |  |  |
| Frail | 1221/3834 | 1.07 (1.05-1.09) | <0.001 |  |  |
| **CRI-I-CRP** | | | | | |
| **Age** |  |  |  | 0.332 | 0.464 |
| 45-60 y | 243/2188 | 1.08 (1.05-1.12) | <0.001 |  |  |
| ≥60 y | 2134/7755 | 1.06 (1.05-1.08) | <0.001 |  |  |
| **Sex** |  |  |  | 0.261 | 0.457 |
| Male | 1390/4926 | 1.09 (1.07-1.10) | <0.001 |  |  |
| Female | 987/5017 | 1.07 (1.05-1.10) | <0.001 |  |  |
| **Smoking** |  |  |  | 0.163 | 0.381 |
| Never | 909/4684 | 1.05 (1.02-1.08) | 0.005 |  |  |
| Former | 1031/3592 | 1.09 (1.07-1.11) | <0.001 |  |  |
| Current | 437/1667 | 1.09 (1.06-1.12) | <0.001 |  |  |
| **Drinking** |  |  |  | 0.540 | 0.54 |
| Never | 681/2923 | 1.07 (1.05-1.10) | <0.001 |  |  |
| Former | 661/1954 | 1.07 (1.05-1.10) | <0.001 |  |  |
| Current | 1035/5066 | 1.09 (1.06-1.11) | <0.001 |  |  |
| **Diabetes** |  |  |  | 0.084 | 0.294 |
| No | 1752/7627 | 1.07 (1.06-1.09) | <0.001 |  |  |
| Yes | 625/2316 | 1.09 (1.07-1.12) | <0.001 |  |  |
| **Hypertension** |  |  |  | 0.055 | 0.294 |
| No | 853/4099 | 1.06 (1.03-1.09) | <0.001 |  |  |
| Yes | 1524/5844 | 1.09 (1.07-1.11) | <0.001 |  |  |
| **Frailty status** |  |  |  | 0.481 | 0.54 |
| Non-frail | 314/2022 | 1.09 (1.04-1.15) | 0.001 |  |  |
| Pre-frail | 842/4087 | 1.05 (1.02-1.08) | 0.007 |  |  |
| Frail | 1221/3834 | 1.06 (1.04-1.07) | <0.001 |  |  |
| **CRI-II-CRP** | | | | | |
| **Age** |  |  |  | 0.338 | 0.473 |
| 45-60 y | 243/2188 | 1.14 (1.08-1.20) | 0.001 |  |  |
| ≥60 y | 2134/7755 | 1.10 (1.07-1.13) | <0.001 |  |  |
| **Sex** |  |  |  | 0.316 | 0.473 |
| Male | 1390/4926 | 1.14 (1.11-1.18) | <0.001 |  |  |
| Female | 987/5017 | 1.12 (1.08-1.16) | <0.001 |  |  |
| **Smoking** |  |  |  | 0.225 | 0.473 |
| Never | 909/4684 | 1.09 (1.04-1.14) | 0.006 |  |  |
| Former | 1031/3592 | 1.16 (1.12-1.20) | <0.001 |  |  |
| Current | 437/1667 | 1.14 (1.09-1.19) | <0.001 |  |  |
| **Drinking** |  |  |  | 0.541 | 0.631 |
| Never | 681/2923 | 1.12 (1.08-1.17) | <0.001 |  |  |
| Former | 661/1954 | 1.12 (1.07-1.17) | <0.001 |  |  |
| Current | 1035/5066 | 1.15 (1.10-1.19) | <0.001 |  |  |
| **Diabetes** |  |  |  | 0.044 | 0.155 |
| No | 1752/7627 | 1.12 (1.09-1.15) | <0.001 |  |  |
| Yes | 625/2316 | 1.17 (1.12-1.22) | <0.001 |  |  |
| **Hypertension** |  |  |  | 0.033 | 0.155 |
| No | 853/4099 | 1.09 (1.05-1.14) | <0.001 |  |  |
| Yes | 1524/5844 | 1.15 (1.12-1.19) | <0.001 |  |  |
| **Frailty status** |  |  |  | 0.704 | 0.704 |
| Non-frail | 314/2022 | 1.14 (1.05-1.23) | 0.004 |  |  |
| Pre-frail | 842/4087 | 1.08 (1.03-1.14) | 0.013 |  |  |
| Frail | 1221/3834 | 1.10 (1.07-1.13) | <0.001 |  |  |
| **LCI-CRP** | | | | | |
| **Age** |  |  |  | 0.295 | 0.351 |
| 45-60 y | 243/2188 | 1.00 (1.00-1.00) | 0.019 |  |  |
| ≥60 y | 2134/7755 | 1.00 (1.00-1.00) | 0.002 |  |  |
| **Sex** |  |  |  | 0.144 | 0.351 |
| Male | 1390/4926 | 1.00 (1.00-1.00) | <0.001 |  |  |
| Female | 987/5017 | 1.00 (1.00-1.00) | 0.002 |  |  |
| **Smoking** |  |  |  | 0.151 | 0.351 |
| Never | 909/4684 | 1.00 (1.00-1.00) | 0.284 |  |  |
| Former | 1031/3592 | 1.00 (1.00-1.00) | <0.001 |  |  |
| Current | 437/1667 | 1.00 (1.00-1.00) | 0.007 |  |  |
| **Drinking** |  |  |  | 0.365 | 0.365 |
| Never | 681/2923 | 1.00 (1.00-1.00) | 0.01 |  |  |
| Former | 661/1954 | 1.00 (1.00-1.00) | <0.001 |  |  |
| Current | 1035/5066 | 1.00 (1.00-1.00) | <0.001 |  |  |
| **Diabetes** |  |  |  | 0.213 | 0.351 |
| No | 1752/7627 | 1.00 (1.00-1.00) | <0.001 |  |  |
| Yes | 625/2316 | 1.00 (1.00-1.00) | <0.001 |  |  |
| **Hypertension** |  |  |  | 0.075 | 0.351 |
| No | 853/4099 | 1.00 (1.00-1.00) | 0.046 |  |  |
| Yes | 1524/5844 | 1.00 (1.00-1.00) | <0.001 |  |  |
| **Frailty status** |  |  |  | 0.301 | 0.351 |
| Non-frail | 314/2022 | 1.00 (1.00-1.00) | 0.002 |  |  |
| Pre-frail | 842/4087 | 1.00 (1.00-1.00) | 0.153 |  |  |
| Frail | 1221/3834 | 1.00 (1.00-1.00) | 0.015 |  |  |
| **RC/HDL-C-CRP** | | | | | |
| **Age** |  |  |  | 0.224 | 0.411 |
| 45-60 y | 243/2188 | 1.51 (1.27-1.79) | <0.001 |  |  |
| ≥60 y | 2134/7755 | 1.33 (1.23-1.45) | <0.001 |  |  |
| **Sex** |  |  |  | 0.387 | 0.452 |
| Male | 1390/4926 | 1.48 (1.35-1.62) | <0.001 |  |  |
| Female | 987/5017 | 1.42 (1.25-1.60) | <0.001 |  |  |
| **Smoking** |  |  |  | 0.286 | 0.411 |
| Never | 909/4684 | 1.25 (1.07-1.45) | 0.042 |  |  |
| Former | 1031/3592 | 1.54 (1.38-1.72) | <0.001 |  |  |
| Current | 437/1667 | 1.52 (1.32-1.75) | <0.001 |  |  |
| **Drinking** |  |  |  | 0.293 | 0.411 |
| Never | 681/2923 | 1.33 (1.17-1.52) | <0.001 |  |  |
| Former | 661/1954 | 1.46 (1.27-1.68) | <0.001 |  |  |
| Current | 1035/5066 | 1.54 (1.37-1.74) | <0.001 |  |  |
| **Diabetes** |  |  |  | 0.529 | 0.529 |
| No | 1752/7627 | 1.45 (1.32-1.59) | <0.001 |  |  |
| Yes | 625/2316 | 1.45 (1.28-1.64) | <0.001 |  |  |
| **Hypertension** |  |  |  | 0.287 | 0.411 |
| No | 853/4099 | 1.38 (1.19-1.60) | <0.001 |  |  |
| Yes | 1524/5844 | 1.49 (1.36-1.62) | <0.001 |  |  |
| **Frailty status** |  |  |  | 0.153 | 0.411 |
| Non-frail | 314/2022 | 1.81 (1.30-2.52) | <0.001 |  |  |
| Pre-frail | 842/4087 | 1.26 (1.07-1.48) | 0.018 |  |  |
| Frail | 1221/3834 | 1.28 (1.17-1.40) | <0.001 |  |  |

Notes: Models were adjusted for age, sex, marital status, education, smoking, drinking, diabetes, hypertension, BMI, and SBP. *P* values for interaction were adjusted for multiple comparisons using the Benjamini-Hochberg method.

Abbreviations: NHANES, National Health and Nutrition Examination Survey; HR, hazard ratio; CI, confidence interval; BH-FDR, Benjamini-Hochberg false discovery rate.

# **Supplementary Table. 11** Subgroup analyses of the non-traditional lipid-inflammatory parameters with all-cause mortality in CHARLS.

| **Subgroups** | **Events/Total** | **HR (95% CI)** | ***P*** | ***P* for interaction** | ***P* for interaction**  **(BH-FDR)** |
| --- | --- | --- | --- | --- | --- |
| **non-HDL-C-CRP** | | | | | |
| **Age** |  |  |  | 0.081 | 0.282 |
| 45-60 y | 279/5430 | 1.00 (1.00-1.00) | <0.001 |  |  |
| ≥60 y | 967/3979 | 1.00 (1.00-1.00) | <0.001 |  |  |
| **Sex** |  |  |  | 0.124 | 0.289 |
| Male | 754/4370 | 1.00 (1.00-1.00) | <0.001 |  |  |
| Female | 492/5039 | 1.00 (1.00-1.00) | 0.006 |  |  |
| **Smoking** |  |  |  | 0.261 | 0.456 |
| Never | 585/5738 | 1.00 (1.00-1.00) | <0.001 |  |  |
| Former | 189/833 | 1.00 (1.00-1.01) | <0.001 |  |  |
| Current | 472/2838 | 1.00 (1.00-1.00) | <0.001 |  |  |
| **Drinking** |  |  |  | 0.912 | 0.912 |
| Never | 664/5748 | 1.00 (1.00-1.00) | <0.001 |  |  |
| Former | 171/806 | 1.00 (1.00-1.00) | <0.001 |  |  |
| Current | 411/2855 | 1.00 (1.00-1.00) | <0.001 |  |  |
| **Diabetes** |  |  |  | 0.602 | 0.843 |
| No | 985/7990 | 1.00 (1.00-1.00) | <0.001 |  |  |
| Yes | 261/1419 | 1.00 (1.00-1.00) | 0.003 |  |  |
| **Hypertension** |  |  |  | <0.001 | 0.003 |
| No | 482/4831 | 1.00 (1.00-1.00) | <0.001 |  |  |
| Yes | 764/4578 | 1.00 (1.00-1.00) | 0.020 |  |  |
| **Frailty status** |  |  |  | 0.833 | 0.912 |
| Non-frail | 390/4192 | 1.00 (1.00-1.00) | 0.067 |  |  |
| Pre-frail | 394/3324 | 1.00 (1.00-1.00) | <0.001 |  |  |
| Frail | 462/1893 | 1.00 (1.00-1.00) | <0.001 |  |  |
| **RC-CRP** | | | | | |
| **Age** |  |  |  | 0.251 | 0.438 |
| 45-60 y | 279/5430 | 1.01 (1.01-1.02) | <0.001 |  |  |
| ≥60 y | 967/3979 | 1.01 (1.01-1.01) | <0.001 |  |  |
| **Sex** |  |  |  | 0.194 | 0.438 |
| Male | 754/4370 | 1.01 (1.01-1.02) | <0.001 |  |  |
| Female | 492/5039 | 1.01 (1.00-1.01) | 0.006 |  |  |
| **Smoking** |  |  |  | 0.315 | 0.441 |
| Never | 585/5738 | 1.01 (1.01-1.01) | <0.001 |  |  |
| Former | 189/833 | 1.02 (1.02-1.03) | <0.001 |  |  |
| Current | 472/2838 | 1.01 (1.01-1.02) | 0.002 |  |  |
| **Drinking** |  |  |  | 0.071 | 0.249 |
| Never | 664/5748 | 1.01 (1.01-1.01) | 0.002 |  |  |
| Former | 171/806 | 1.02 (1.01-1.03) | <0.001 |  |  |
| Current | 411/2855 | 1.01 (1.00-1.02) | <0.001 |  |  |
| **Diabetes** |  |  |  | 0.825 | 0.885 |
| No | 985/7990 | 1.01 (1.01-1.01) | <0.001 |  |  |
| Yes | 261/1419 | 1.01 (1.01-1.02) | <0.001 |  |  |
| **Hypertension** |  |  |  | 0.008 | 0.053 |
| No | 482/4831 | 1.02 (1.02-1.02) | <0.001 |  |  |
| Yes | 764/4578 | 1.01 (1.00-1.01) | 0.021 |  |  |
| **Frailty status** |  |  |  | 0.885 | 0.885 |
| Non-frail | 390/4192 | 1.01 (1.00-1.01) | 0.091 |  |  |
| Pre-frail | 394/3324 | 1.01 (1.01-1.02) | <0.001 |  |  |
| Frail | 462/1893 | 1.01 (1.01-1.02) | <0.001 |  |  |
| **AIP-CRP** | | | | | |
| **Age** |  |  |  | 0.072 | 0.251 |
| 45-60 y | 279/5430 | 2.55 (1.62-4.00) | <0.001 |  |  |
| ≥60 y | 967/3979 | 1.88 (1.49-2.36) | <0.001 |  |  |
| **Sex** |  |  |  | 0.947 | 0.997 |
| Male | 754/4370 | 1.89 (1.41-2.54) | <0.001 |  |  |
| Female | 492/5039 | 1.87 (1.39-2.50) | 0.002 |  |  |
| **Smoking** |  |  |  | 0.735 | 0.997 |
| Never | 585/5738 | 1.73 (1.28-2.34) | 0.007 |  |  |
| Former | 189/833 | 3.51 (1.96-6.30) | <0.001 |  |  |
| Current | 472/2838 | 2.02 (1.45-2.82) | <0.001 |  |  |
| **Drinking** |  |  |  | 0.174 | 0.406 |
| Never | 664/5748 | 2.02 (1.53-2.65) | <0.001 |  |  |
| Former | 171/806 | 3.43 (2.16-5.46) | <0.001 |  |  |
| Current | 411/2855 | 1.54 (1.01-2.34) | 0.086 |  |  |
| **Diabetes** |  |  |  | 0.877 | 0.997 |
| No | 985/7990 | 1.77 (1.34-2.33) | 0.002 |  |  |
| Yes | 261/1419 | 1.82 (1.31-2.52) | 0.003 |  |  |
| **Hypertension** |  |  |  | 0.017 | 0.118 |
| No | 482/4831 | 3.25 (2.38-4.43) | <0.001 |  |  |
| Yes | 764/4578 | 1.43 (1.09-1.89) | 0.063 |  |  |
| **Frailty status** |  |  |  | 0.997 | 0.997 |
| Non-frail | 390/4192 | 1.70 (1.09-2.66) | 0.055 |  |  |
| Pre-frail | 394/3324 | 1.96 (1.42-2.70) | <0.001 |  |  |
| Frail | 462/1893 | 1.96 (1.39-2.78) | 0.006 |  |  |
| **AC-CRP** | | | | | |
| **Age** |  |  |  | 0.114 | 0.398 |
| 45-60 y | 279/5430 | 1.12 (1.06-1.18) | <0.001 |  |  |
| ≥60 y | 967/3979 | 1.09 (1.06-1.11) | <0.001 |  |  |
| **Sex** |  |  |  | 0.210 | 0.489 |
| Male | 754/4370 | 1.10 (1.07-1.14) | <0.001 |  |  |
| Female | 492/5039 | 1.07 (1.03-1.10) | 0.011 |  |  |
| **Smoking** |  |  |  | 0.375 | 0.657 |
| Never | 585/5738 | 1.08 (1.04-1.12) | <0.001 |  |  |
| Former | 189/833 | 1.19 (1.12-1.28) | <0.001 |  |  |
| Current | 472/2838 | 1.08 (1.04-1.13) | <0.001 |  |  |
| **Drinking** |  |  |  | 0.854 | 0.854 |
| Never | 664/5748 | 1.08 (1.05-1.12) | <0.001 |  |  |
| Former | 171/806 | 1.12 (1.06-1.20) | <0.001 |  |  |
| Current | 411/2855 | 1.09 (1.04-1.14) | 0.003 |  |  |
| **Diabetes** |  |  |  | 0.842 | 0.854 |
| No | 985/7990 | 1.08 (1.05-1.11) | <0.001 |  |  |
| Yes | 261/1419 | 1.07 (1.03-1.12) | 0.003 |  |  |
| **Hypertension** |  |  |  | 0.001 | 0.007 |
| No | 482/4831 | 1.17 (1.13-1.21) | <0.001 |  |  |
| Yes | 764/4578 | 1.04 (1.01-1.08) | 0.070 |  |  |
| **Frailty status** |  |  |  | 0.776 | 0.854 |
| Non-frail | 390/4192 | 1.05 (1.00-1.10) | 0.171 |  |  |
| Pre-frail | 394/3324 | 1.09 (1.05-1.13) | <0.001 |  |  |
| Frail | 462/1893 | 1.10 (1.06-1.15) | <0.001 |  |  |
| **CRI-I-CRP** | | | | | |
| **Age** |  |  |  | 0.090 | 0.315 |
| 45-60 y | 279/5430 | 1.10 (1.05-1.14) | <0.001 |  |  |
| ≥60 y | 967/3979 | 1.07 (1.05-1.09) | <0.001 |  |  |
| **Sex** |  |  |  | 0.177 | 0.413 |
| Male | 754/4370 | 1.08 (1.06-1.11) | <0.001 |  |  |
| Female | 492/5039 | 1.05 (1.02-1.08) | 0.008 |  |  |
| **Smoking** |  |  |  | 0.286 | 0.501 |
| Never | 585/5738 | 1.06 (1.04-1.09) | <0.001 |  |  |
| Former | 189/833 | 1.15 (1.09-1.21) | <0.001 |  |  |
| Current | 472/2838 | 1.07 (1.04-1.10) | <0.001 |  |  |
| **Drinking** |  |  |  | 0.874 | 0.874 |
| Never | 664/5748 | 1.07 (1.04-1.09) | <0.001 |  |  |
| Former | 171/806 | 1.09 (1.04-1.15) | <0.001 |  |  |
| Current | 411/2855 | 1.07 (1.04-1.11) | <0.001 |  |  |
| **Diabetes** |  |  |  | 0.659 | 0.874 |
| No | 985/7990 | 1.07 (1.05-1.09) | <0.001 |  |  |
| Yes | 261/1419 | 1.05 (1.02-1.09) | 0.003 |  |  |
| **Hypertension** |  |  |  | <0.001 | 0.004 |
| No | 482/4831 | 1.13 (1.10-1.16) | <0.001 |  |  |
| Yes | 764/4578 | 1.04 (1.01-1.06) | 0.039 |  |  |
| **Frailty status** |  |  |  | 0.780 | 0.874 |
| Non-frail | 390/4192 | 1.04 (1.00-1.08) | 0.117 |  |  |
| Pre-frail | 394/3324 | 1.07 (1.04-1.10) | <0.001 |  |  |
| Frail | 462/1893 | 1.08 (1.05-1.11) | <0.001 |  |  |
| **CRI-II-CRP** | | | | | |
| **Age** |  |  |  | 0.056 | 0.194 |
| 45-60 y | 279/5430 | 1.17 (1.09-1.25) | <0.001 |  |  |
| ≥60 y | 967/3979 | 1.10 (1.07-1.14) | <0.001 |  |  |
| **Sex** |  |  |  | 0.198 | 0.462 |
| Male | 754/4370 | 1.13 (1.09-1.18) | <0.001 |  |  |
| Female | 492/5039 | 1.08 (1.03-1.13) | 0.012 |  |  |
| **Smoking** |  |  |  | 0.420 | 0.734 |
| Never | 585/5738 | 1.10 (1.05-1.15) | <0.001 |  |  |
| Former | 189/833 | 1.22 (1.12-1.33) | <0.001 |  |  |
| Current | 472/2838 | 1.11 (1.06-1.16) | <0.001 |  |  |
| **Drinking** |  |  |  | 0.948 | 0.948 |
| Never | 664/5748 | 1.11 (1.06-1.15) | <0.001 |  |  |
| Former | 171/806 | 1.12 (1.03-1.22) | 0.005 |  |  |
| Current | 411/2855 | 1.12 (1.05-1.18) | 0.002 |  |  |
| **Diabetes** |  |  |  | 0.683 | 0.891 |
| No | 985/7990 | 1.11 (1.07-1.15) | <0.001 |  |  |
| Yes | 261/1419 | 1.08 (1.02-1.14) | 0.012 |  |  |
| **Hypertension** |  |  |  | <0.001 | 0.003 |
| No | 482/4831 | 1.22 (1.16-1.27) | <0.001 |  |  |
| Yes | 764/4578 | 1.05 (1.01-1.09) | 0.093 |  |  |
| **Frailty status** |  |  |  | 0.764 | 0.891 |
| Non-frail | 390/4192 | 1.06 (1.00-1.13) | 0.185 |  |  |
| Pre-frail | 394/3324 | 1.12 (1.07-1.17) | <0.001 |  |  |
| Frail | 462/1893 | 1.13 (1.07-1.19) | <0.001 |  |  |
| **LCI-CRP** | | | | | |
| **Age** |  |  |  | 0.635 | 0.929 |
| 45-60 y | 279/5430 | 1.00 (1.00-1.00) | 0.012 |  |  |
| ≥60 y | 967/3979 | 1.00 (1.00-1.00) | <0.001 |  |  |
| **Sex** |  |  |  | 0.454 | 0.929 |
| Male | 754/4370 | 1.00 (1.00-1.00) | <0.001 |  |  |
| Female | 492/5039 | 1.00 (1.00-1.00) | 0.008 |  |  |
| **Smoking** |  |  |  | 0.856 | 0.929 |
| Never | 585/5738 | 1.00 (1.00-1.00) | 0.005 |  |  |
| Former | 189/833 | 1.00 (1.00-1.00) | <0.001 |  |  |
| Current | 472/2838 | 1.00 (1.00-1.00) | 0.005 |  |  |
| **Drinking** |  |  |  | 0.752 | 0.929 |
| Never | 664/5748 | 1.00 (1.00-1.00) | <0.001 |  |  |
| Former | 171/806 | 1.00 (1.00-1.00) | <0.001 |  |  |
| Current | 411/2855 | 1.00 (1.00-1.00) | 0.034 |  |  |
| **Diabetes** |  |  |  | 0.754 | 0.929 |
| No | 985/7990 | 1.00 (1.00-1.00) | 0.002 |  |  |
| Yes | 261/1419 | 1.00 (1.00-1.00) | 0.007 |  |  |
| **Hypertension** |  |  |  | 0.007 | 0.052 |
| No | 482/4831 | 1.00 (1.00-1.00) | <0.001 |  |  |
| Yes | 764/4578 | 1.00 (1.00-1.00) | 0.054 |  |  |
| **Frailty status** |  |  |  | 0.929 | 0.929 |
| Non-frail | 390/4192 | 1.00 (1.00-1.00) | 0.138 |  |  |
| Pre-frail | 394/3324 | 1.00 (1.00-1.00) | <0.001 |  |  |
| Frail | 462/1893 | 1.00 (1.00-1.00) | 0.010 |  |  |
| **RC/HDL-C-CRP** | | | | | |
| **Age** |  |  |  | 0.268 | 0.548 |
| 45-60 y | 279/5430 | 1.54 (1.21-1.95) | 0.001 |  |  |
| ≥60 y | 967/3979 | 1.42 (1.26-1.60) | <0.001 |  |  |
| **Sex** |  |  |  | 0.313 | 0.548 |
| Male | 754/4370 | 1.51 (1.30-1.75) | <0.001 |  |  |
| Female | 492/5039 | 1.31 (1.12-1.54) | 0.012 |  |  |
| **Smoking** |  |  |  | 0.548 | 0.767 |
| Never | 585/5738 | 1.33 (1.13-1.57) | 0.010 |  |  |
| Former | 189/833 | 2.11 (1.60-2.79) | <0.001 |  |  |
| Current | 472/2838 | 1.44 (1.21-1.72) | 0.001 |  |  |
| **Drinking** |  |  |  | 0.040 | 0.139 |
| Never | 664/5748 | 1.37 (1.18-1.59) | 0.002 |  |  |
| Former | 171/806 | 2.02 (1.60-2.55) | <0.001 |  |  |
| Current | 411/2855 | 1.38 (1.11-1.72) | 0.009 |  |  |
| **Diabetes** |  |  |  | 0.765 | 0.893 |
| No | 985/7990 | 1.37 (1.18-1.58) | 0.002 |  |  |
| Yes | 261/1419 | 1.44 (1.22-1.71) | 0.001 |  |  |
| **Hypertension** |  |  |  | 0.013 | 0.090 |
| No | 482/4831 | 1.92 (1.63-2.25) | <0.001 |  |  |
| Yes | 764/4578 | 1.22 (1.06-1.41) | 0.062 |  |  |
| **Frailty status** |  |  |  | 0.997 | 0.997 |
| Non-frail | 390/4192 | 1.31 (1.04-1.65) | 0.074 |  |  |
| Pre-frail | 394/3324 | 1.45 (1.22-1.71) | <0.001 |  |  |
| Frail | 462/1893 | 1.45 (1.21-1.73) | 0.002 |  |  |

Notes: Models were adjusted for age, sex, marital status, education, smoking, drinking, diabetes, hypertension, BMI, and SBP. *P* values for interaction were adjusted for multiple comparisons using the Benjamini-Hochberg method.

Abbreviations: CHARLS, China Health and Retirement Longitudinal Study; HR, hazard ratio; CI, confidence interval; BH-FDR, Benjamini-Hochberg false discovery rate.

# **Supplementary Table. 12** Sensitivity analyses of additional control variables specific to the database.

| **Parameters** | **NHANES** | | **CHARLS** | |
| --- | --- | --- | --- | --- |
|  | **HR (95% CI)** | ***P*** | **HR (95% CI)** | ***P*** |
| **non-HDL-C-CRP** |  |  |  |  |
| Per 1-SD | 1.22 (1.15-1.28) | <0.001 | 1.15 (1.09-1.22) | <0.001 |
| Q1 | Ref |  | Ref |  |
| Q2 | 1.11 (0.98-1.26) | 0.111 | 1.04 (0.82-1.32) | 0.722 |
| Q3 | 1.18 (1.02-1.38) | 0.031 | 1.38 (1.06-1.80) | 0.017 |
| Q4 | 1.50 (1.31-1.72) | <0.001 | 1.54 (1.22-1.95) | <0.001 |
| *P_trend_* | <0.001 |  | <0.001 |  |
| **RC-CRP** |  |  |  |  |
| Per 1-SD | 1.20 (1.14-1.26) | <0.001 | 1.15 (1.08-1.22) | <0.001 |
| Q1 | Ref |  | Ref |  |
| Q2 | 1.03 (0.87-1.23) | 0.725 | 1.11 (0.87-1.40) | 0.398 |
| Q3 | 1.11 (0.97-1.28) | 0.127 | 1.41 (1.09-1.82) | 0.008 |
| Q4 | 1.39 (1.22-1.58) | <0.001 | 1.58 (1.21-2.07) | <0.001 |
| *P_trend_* | <0.001 |  | <0.001 |  |
| **AIP-CRP** |  |  |  |  |
| Per 1-SD | 1.20 (1.14-1.27) | <0.001 | 1.13 (1.06-1.20) | <0.001 |
| Q1 | Ref |  | Ref |  |
| Q2 | 0.82 (0.71-0.95) | 0.006 | 0.89 (0.71-1.13) | 0.340 |
| Q3 | 0.91 (0.80-1.04) | 0.167 | 0.96 (0.75-1.23) | 0.753 |
| Q4 | 1.17 (1.02-1.34) | 0.022 | 1.16 (0.88-1.52) | 0.298 |
| *P_trend_* | 0.004 |  | 0.208 |  |
| **AC-CRP** |  |  |  |  |
| Per 1-SD | 1.23 (1.17-1.29) | <0.001 | 1.14 (1.07-1.20) | <0.001 |
| Q1 | Ref |  | Ref |  |
| Q2 | 1.04 (0.92-1.17) | 0.523 | 1.07 (0.84-1.37) | 0.557 |
| Q3 | 1.18 (1.01-1.38) | 0.040 | 1.24 (0.94-1.64) | 0.124 |
| Q4 | 1.44 (1.28-1.63) | <0.001 | 1.47 (1.13-1.90) | 0.004 |
| *P_trend_* | <0.001 |  | 0.001 |  |
| **CRI-I-CRP** |  |  |  |  |
| Per 1-SD | 1.24 (1.18-1.31) | <0.001 | 1.15 (1.08-1.21) | <0.001 |
| Q1 | Ref |  | Ref |  |
| Q2 | 1.12 (0.98-1.27) | 0.103 | 1.13 (0.90-1.42) | 0.286 |
| Q3 | 1.19 (1.01-1.40) | 0.035 | 1.31 (1.00-1.71) | 0.052 |
| Q4 | 1.53 (1.34-1.74) | <0.001 | 1.59 (1.24-2.05) | <0.001 |
| *P_trend_* | <0.001 |  | <0.001 |  |
| **CRI-II-CRP** |  |  |  |  |
| Per 1-SD | 1.23 (1.16-1.29) | <0.001 | 1.13 (1.07-1.20) | <0.001 |
| Q1 | Ref |  | Ref |  |
| Q2 | 1.10 (0.97-1.25) | 0.131 | 1.05 (0.82-1.33) | 0.705 |
| Q3 | 1.16 (0.99-1.36) | 0.068 | 1.31 (0.99-1.72) | 0.054 |
| Q4 | 1.46 (1.29-1.66) | <0.001 | 1.44 (1.13-1.84) | 0.003 |
| *P_trend_* | <0.001 |  | <0.001 |  |
| **LCI-CRP** |  |  |  |  |
| Per 1-SD | 1.16 (1.09-1.23) | <0.001 | 1.12 (1.06-1.19) | <0.001 |
| Q1 | Ref |  | Ref |  |
| Q2 | 1.00 (0.89-1.12) | 0.939 | 0.89 (0.71-1.12) | 0.320 |
| Q3 | 1.07 (0.94-1.22) | 0.292 | 1.01 (0.77-1.34) | 0.922 |
| Q4 | 1.17 (1.03-1.32) | 0.015 | 1.28 (0.99-1.64) | 0.055 |
| *P_trend_* | 0.005 |  | 0.025 |  |
| **RC/HDL-C-CRP** |  |  |  |  |
| Per 1-SD | 1.20 (1.14-1.27) | <0.001 | 1.14 (1.07-1.21) | <0.001 |
| Q1 | Ref |  | Ref |  |
| Q2 | 0.95 (0.82-1.10) | 0.525 | 1.15 (0.91-1.45) | 0.240 |
| Q3 | 1.07 (0.93-1.23) | 0.378 | 1.32 (1.01-1.73) | 0.045 |
| Q4 | 1.31 (1.15-1.49) | <0.001 | 1.50 (1.15-1.96) | 0.003 |
| *P_trend_* | <0.001 |  | 0.002 |  |

Note: NHANES models were adjusted for age, sex, race, marital status, education, PIR, smoking, drinking, diabetes, hypertension, BMI, and SBP; CHARLS models were adjusted for age, sex, residence, marital status, education, smoking, drinking, diabetes, hypertension, BMI, and SBP.

Abbreviations: NHANES, National Health and Nutrition Examination Survey; CHARLS, China Health and Retirement Longitudinal Study; HR, hazard ratio; CI, confidence interval; Ref, reference.

# **Supplementary Table. 13** Sensitivity analyses of additional control other possible clinically relevant confounders.

| **Parameters** | **Model 4** | | **Model 5** | | **Model 6** | |
| --- | --- | --- | --- | --- | --- | --- |
|  | **HR (95% CI)** | ***P*** | **HR (95% CI)** | ***P*** | **HR (95% CI)** | ***P*** |
| **NHANES** | | | | | | |
| **non-HDL-C-CRP** |  |  |  |  |  |  |
| Per 1-SD | 1.22 (1.16-1.29) | <0.001 | 1.22 (1.16-1.28) | <0.001 | 1.22 (1.15-1.28) | <0.001 |
| Q1 | Ref |  | Ref |  | Ref |  |
| Q2 | 1.13 (1.00-1.28) | 0.049 | 1.13 (1.00-1.27) | 0.055 | 1.13 (1.00-1.28) | 0.054 |
| Q3 | 1.23 (1.06-1.43) | 0.007 | 1.23 (1.06-1.44) | 0.007 | 1.24 (1.06-1.44) | 0.006 |
| Q4 | 1.56 (1.35-1.79) | <0.001 | 1.53 (1.33-1.76) | <0.001 | 1.52 (1.32-1.75) | <0.001 |
| *P_trend_* | <0.001 |  | <0.001 |  | <0.001 |  |
| **RC-CRP** |  |  |  |  |  |  |
| Per 1-SD | 1.21 (1.15-1.27) | <0.001 | 1.20 (1.14-1.26) | <0.001 | 1.19 (1.13-1.25) | <0.001 |
| Q1 | Ref |  | Ref |  | Ref |  |
| Q2 | 1.05 (0.88-1.24) | 0.592 | 1.04 (0.88-1.23) | 0.661 | 1.04 (0.88-1.23) | 0.646 |
| Q3 | 1.15 (1.00-1.31) | 0.045 | 1.15 (1.01-1.32) | 0.041 | 1.15 (1.01-1.32) | 0.039 |
| Q4 | 1.44 (1.26-1.64) | <0.001 | 1.41 (1.24-1.61) | <0.001 | 1.40 (1.23-1.60) | <0.001 |
| *P_trend_* | <0.001 |  | <0.001 |  | <0.001 |  |
| **AIP-CRP** |  |  |  |  |  |  |
| Per 1-SD | 1.21 (1.15-1.27) | <0.001 | 1.20 (1.13-1.26) | <0.001 | 1.19 (1.13-1.26) | <0.001 |
| Q1 | Ref |  | Ref |  | Ref |  |
| Q2 | 0.84 (0.73-0.96) | 0.014 | 0.83 (0.72-0.96) | 0.009 | 0.84 (0.73-0.96) | 0.013 |
| Q3 | 0.94 (0.83-1.06) | 0.322 | 0.94 (0.83-1.06) | 0.310 | 0.94 (0.82-1.06) | 0.305 |
| Q4 | 1.22 (1.07-1.39) | 0.003 | 1.20 (1.05-1.37) | 0.007 | 1.19 (1.05-1.36) | 0.008 |
| *P_trend_* | <0.001 |  | <0.001 |  | 0.001 |  |
| **AC-CRP** |  |  |  |  |  |  |
| Per 1-SD | 1.23 (1.17-1.30) | <0.001 | 1.22 (1.16-1.29) | <0.001 | 1.22 (1.16-1.29) | <0.001 |
| Q1 | Ref |  | Ref |  | Ref |  |
| Q2 | 1.06 (0.94-1.19) | 0.332 | 1.05 (0.94-1.18) | 0.358 | 1.06 (0.95-1.19) | 0.301 |
| Q3 | 1.23 (1.05-1.43) | 0.008 | 1.23 (1.05-1.43) | 0.009 | 1.23 (1.05-1.44) | 0.008 |
| Q4 | 1.49 (1.32-1.69) | <0.001 | 1.46 (1.29-1.66) | <0.001 | 1.46 (1.28-1.66) | <0.001 |
| *P_trend_* | <0.001 |  | <0.001 |  | <0.001 |  |
| **CRI-I-CRP** |  |  |  |  |  |  |
| Per 1-SD | 1.25 (1.19-1.31) | <0.001 | 1.24 (1.18-1.30) | <0.001 | 1.23 (1.17-1.30) | <0.001 |
| Q1 | Ref |  | Ref |  | Ref |  |
| Q2 | 1.13 (1.00-1.28) | 0.055 | 1.13 (1.00-1.27) | 0.060 | 1.13 (1.00-1.28) | 0.059 |
| Q3 | 1.23 (1.05-1.45) | 0.011 | 1.23 (1.04-1.45) | 0.014 | 1.23 (1.05-1.45) | 0.012 |
| Q4 | 1.58 (1.39-1.81) | <0.001 | 1.55 (1.36-1.78) | <0.001 | 1.55 (1.35-1.77) | <0.001 |
| *P_trend_* | <0.001 |  | <0.001 |  | <0.001 |  |
| **CRI-II-CRP** |  |  |  |  |  |  |
| Per 1-SD | 1.23 (1.17-1.30) | <0.001 | 1.23 (1.16-1.29) | <0.001 | 1.22 (1.16-1.29) | <0.001 |
| Q1 | Ref |  | Ref |  | Ref |  |
| Q2 | 1.13 (1.01-1.28) | 0.038 | 1.12 (1.00-1.27) | 0.056 | 1.13 (1.00-1.28) | 0.047 |
| Q3 | 1.21 (1.03-1.43) | 0.018 | 1.21 (1.03-1.43) | 0.022 | 1.22 (1.03-1.44) | 0.019 |
| Q4 | 1.53 (1.35-1.74) | <0.001 | 1.50 (1.32-1.71) | <0.001 | 1.50 (1.31-1.71) | <0.001 |
| *P_trend_* | <0.001 |  | <0.001 |  | <0.001 |  |
| **LCI-CRP** |  |  |  |  |  |  |
| Per 1-SD | 1.17 (1.10-1.24) | <0.001 | 1.16 (1.09-1.23) | <0.001 | 1.16 (1.09-1.23) | <0.001 |
| Q1 | Ref |  | Ref |  | Ref |  |
| Q2 | 1.03 (0.92-1.15) | 0.613 | 1.03 (0.92-1.15) | 0.664 | 1.03 (0.92-1.16) | 0.552 |
| Q3 | 1.12 (1.00-1.26) | 0.054 | 1.12 (1.00-1.26) | 0.057 | 1.12 (1.00-1.25) | 0.052 |
| Q4 | 1.23 (1.10-1.39) | <0.001 | 1.21 (1.07-1.37) | 0.002 | 1.20 (1.07-1.36) | 0.003 |
| *P_trend_* | <0.001 |  | <0.001 |  | <0.001 |  |
| **RC/HDL-C-CRP** |  |  |  |  |  |  |
| Per 1-SD | 1.21 (1.15-1.27) | <0.001 | 1.20 (1.14-1.26) | <0.001 | 1.19 (1.13-1.25) | <0.001 |
| Q1 | Ref |  | Ref |  | Ref |  |
| Q2 | 0.98 (0.85-1.13) | 0.787 | 0.97 (0.84-1.12) | 0.680 | 0.98 (0.85-1.12) | 0.749 |
| Q3 | 1.10 (0.96-1.26) | 0.179 | 1.10 (0.96-1.26) | 0.188 | 1.10 (0.96-1.26) | 0.171 |
| Q4 | 1.36 (1.20-1.54) | <0.001 | 1.33 (1.17-1.51) | <0.001 | 1.33 (1.17-1.51) | <0.001 |
| *P_trend_* | <0.001 |  | <0.001 |  | <0.001 |  |
| **CHARLS** | | | | | | |
| **non-HDL-C-CRP** |  |  |  |  |  |  |
| Per 1-SD | 1.16 (1.09-1.22) | <0.001 | 1.16 (1.10-1.22) | <0.001 | 1.16 (1.10-1.22) | <0.001 |
| Q1 | Ref |  | Ref |  | Ref |  |
| Q2 | 1.04 (0.82-1.33) | 0.723 | 1.06 (0.84-1.35) | 0.628 | 1.07 (0.84-1.35) | 0.595 |
| Q3 | 1.36 (1.04-1.79) | 0.025 | 1.37 (1.04-1.80) | 0.023 | 1.38 (1.05-1.81) | 0.021 |
| Q4 | 1.53 (1.21-1.95) | <0.001 | 1.53 (1.20-1.95) | <0.001 | 1.52 (1.20-1.94) | <0.001 |
| *P_trend_* | <0.001 |  | <0.001 |  | <0.001 |  |
| **RC-CRP** |  |  |  |  |  |  |
| Per 1-SD | 1.15 (1.08-1.23) | <0.001 | 1.16 (1.09-1.23) | <0.001 | 1.16 (1.09-1.23) | <0.001 |
| Q1 | Ref |  | Ref |  | Ref |  |
| Q2 | 1.10 (0.86-1.40) | 0.436 | 1.11 (0.87-1.41) | 0.417 | 1.12 (0.87-1.42) | 0.381 |
| Q3 | 1.40 (1.08-1.83) | 0.011 | 1.41 (1.09-1.82) | 0.010 | 1.41 (1.09-1.83) | 0.008 |
| Q4 | 1.56 (1.18-2.07) | 0.002 | 1.54 (1.17-2.03) | 0.002 | 1.54 (1.17-2.03) | 0.002 |
| *P_trend_* | <0.001 |  | <0.001 |  | <0.001 |  |
| **AIP-CRP** |  |  |  |  |  |  |
| Per 1-SD | 1.13 (1.06-1.20) | <0.001 | 1.13 (1.07-1.20) | <0.001 | 1.13 (1.07-1.20) | <0.001 |
| Q1 | Ref |  | Ref |  | Ref |  |
| Q2 | 0.89 (0.71-1.12) | 0.307 | 0.91 (0.73-1.12) | 0.373 | 0.90 (0.73-1.12) | 0.358 |
| Q3 | 0.96 (0.75-1.24) | 0.771 | 0.98 (0.77-1.25) | 0.859 | 0.98 (0.77-1.25) | 0.868 |
| Q4 | 1.15 (0.87-1.52) | 0.322 | 1.16 (0.89-1.50) | 0.268 | 1.15 (0.89-1.49) | 0.277 |
| *P_trend_* | 0.220 |  | 0.189 |  | 0.193 |  |
| **AC-CRP** |  |  |  |  |  |  |
| Per 1-SD | 1.14 (1.08-1.21) | <0.001 | 1.14 (1.08-1.21) | <0.001 | 1.14 (1.08-1.21) | <0.001 |
| Q1 | Ref |  | Ref |  | Ref |  |
| Q2 | 1.07 (0.84-1.37) | 0.588 | 1.08 (0.85-1.38) | 0.519 | 1.09 (0.85-1.39) | 0.487 |
| Q3 | 1.23 (0.93-1.63) | 0.152 | 1.23 (0.93-1.63) | 0.146 | 1.24 (0.94-1.64) | 0.132 |
| Q4 | 1.45 (1.11-1.91) | 0.007 | 1.45 (1.11-1.88) | 0.006 | 1.45 (1.11-1.88) | 0.006 |
| *P_trend_* | 0.002 |  | 0.002 |  | 0.002 |  |
| **CRI-I-CRP** |  |  |  |  |  |  |
| Per 1-SD | 1.15 (1.09-1.22) | <0.001 | 1.15 (1.09-1.22) | <0.001 | 1.15 (1.09-1.22) | <0.001 |
| Q1 | Ref |  | Ref |  | Ref |  |
| Q2 | 1.13 (0.90-1.43) | 0.292 | 1.15 (0.91-1.45) | 0.252 | 1.15 (0.91-1.45) | 0.240 |
| Q3 | 1.29 (0.98-1.70) | 0.070 | 1.29 (0.98-1.70) | 0.067 | 1.30 (0.99-1.71) | 0.060 |
| Q4 | 1.58 (1.22-2.06) | <0.001 | 1.58 (1.22-2.04) | <0.001 | 1.57 (1.22-2.03) | <0.001 |
| *P_trend_* | <0.001 |  | <0.001 |  | <0.001 |  |
| **CRI-II-CRP** |  |  |  |  |  |  |
| Per 1-SD | 1.14 (1.07-1.20) | <0.001 | 1.14 (1.08-1.21) | <0.001 | 1.14 (1.08-1.21) | <0.001 |
| Q1 | Ref |  | Ref |  | Ref |  |
| Q2 | 1.04 (0.81-1.32) | 0.778 | 1.05 (0.82-1.34) | 0.680 | 1.06 (0.83-1.35) | 0.639 |
| Q3 | 1.30 (0.98-1.72) | 0.071 | 1.30 (0.98-1.72) | 0.065 | 1.31 (0.99-1.73) | 0.059 |
| Q4 | 1.43 (1.11-1.84) | 0.006 | 1.42 (1.11-1.83) | 0.006 | 1.42 (1.11-1.82) | 0.006 |
| *P_trend_* | 0.001 |  | 0.001 |  | 0.001 |  |
| **LCI-CRP** |  |  |  |  |  |  |
| Per 1-SD | 1.12 (1.06-1.19) | <0.001 | 1.12 (1.06-1.19) | <0.001 | 1.12 (1.06-1.19) | <0.001 |
| Q1 | Ref |  | Ref |  | Ref |  |
| Q2 | 0.88 (0.71-1.11) | 0.290 | 0.89 (0.71-1.12) | 0.310 | 0.89 (0.71-1.12) | 0.337 |
| Q3 | 1.00 (0.75-1.33) | 0.983 | 1.01 (0.76-1.34) | 0.933 | 1.02 (0.77-1.35) | 0.907 |
| Q4 | 1.27 (0.97-1.65) | 0.080 | 1.28 (0.99-1.65) | 0.064 | 1.28 (0.99-1.65) | 0.062 |
| *P_trend_* | 0.038 |  | 0.026 |  | 0.026 |  |
| **RC/HDL-C-CRP** |  |  |  |  |  |  |
| Per 1-SD | 1.14 (1.07-1.21) | <0.001 | 1.14 (1.07-1.21) | <0.001 | 1.14 (1.07-1.21) | <0.001 |
| Q1 | Ref |  | Ref |  | Ref |  |
| Q2 | 1.15 (0.91-1.46) | 0.255 | 1.15 (0.90-1.46) | 0.258 | 1.16 (0.91-1.47) | 0.231 |
| Q3 | 1.32 (0.99-1.74) | 0.055 | 1.31 (1.00-1.71) | 0.052 | 1.31 (1.01-1.72) | 0.045 |
| Q4 | 1.49 (1.12-1.98) | 0.006 | 1.48 (1.12-1.95) | 0.006 | 1.48 (1.12-1.95) | 0.006 |
| *P_trend_* | 0.005 |  | 0.004 |  | 0.004 |  |

Note: Model 4 was adjusted for age, sex, marital status, education, smoking, drinking, diabetes, hypertension, heart disease, BMI, and SBP. Model 5 was adjusted for Model 4 + stroke. Model 6 was adjusted for Model 5 + cancer.

Abbreviations: NHANES, National Health and Nutrition Examination Survey; CHARLS, China Health and Retirement Longitudinal Study; HR, hazard ratio; CI, confidence interval; Ref, reference.

# **Supplementary Table. 14** Sensitivity analyses excluding participants who died in the first year.

| **Parameters** | **NHANES** | | **CHARLS** | |
| --- | --- | --- | --- | --- |
|  | **HR (95% CI)** | ***P*** | **HR (95% CI)** | ***P*** |
| **non-HDL-C-CRP** |  |  |  |  |
| Per 1-SD | 1.17 (1.12-1.23) | <0.001 | 1.12 (1.06-1.19) | <0.001 |
| Q1 | Ref |  | Ref |  |
| Q2 | 1.11 (0.96-1.27) | 0.147 | 1.06 (0.83-1.35) | 0.645 |
| Q3 | 1.19 (1.02-1.39) | 0.025 | 1.41 (1.07-1.85) | 0.013 |
| Q4 | 1.48 (1.28-1.71) | <0.001 | 1.49 (1.18-1.89) | <0.001 |
| *P_trend_* | <0.001 |  | <0.001 |  |
| **RC-CRP** |  |  |  |  |
| Per 1-SD | 1.17 (1.11-1.22) | <0.001 | 1.12 (1.05-1.20) | <0.001 |
| Q1 | Ref |  | Ref |  |
| Q2 | 1.02 (0.86-1.22) | 0.800 | 1.17 (0.92-1.49) | 0.204 |
| Q3 | 1.14 (0.99-1.31) | 0.078 | 1.44 (1.11-1.87) | 0.005 |
| Q4 | 1.40 (1.23-1.60) | <0.001 | 1.56 (1.20-2.04) | 0.001 |
| *P_trend_* | <0.001 |  | <0.001 |  |
| **AIP-CRP** |  |  |  |  |
| Per 1-SD | 1.17 (1.11-1.23) | <0.001 | 1.10 (1.03-1.17) | 0.003 |
| Q1 | Ref |  | Ref |  |
| Q2 | 0.80 (0.70-0.93) | 0.003 | 0.90 (0.71-1.15) | 0.411 |
| Q3 | 0.93 (0.82-1.05) | 0.237 | 0.98 (0.76-1.27) | 0.866 |
| Q4 | 1.16 (1.01-1.33) | 0.037 | 1.15 (0.87-1.51) | 0.332 |
| *P_trend_* | 0.005 |  | 0.228 |  |
| **AC-CRP** |  |  |  |  |
| Per 1-SD | 1.18 (1.13-1.24) | <0.001 | 1.10 (1.04-1.17) | <0.001 |
| Q1 | Ref |  | Ref |  |
| Q2 | 1.06 (0.93-1.20) | 0.378 | 1.11 (0.86-1.42) | 0.429 |
| Q3 | 1.17 (1.00-1.37) | 0.056 | 1.26 (0.95-1.67) | 0.106 |
| Q4 | 1.43 (1.24-1.63) | <0.001 | 1.45 (1.12-1.88) | 0.004 |
| *P_trend_* | <0.001 |  | 0.001 |  |
| **CRI-I-CRP** |  |  |  |  |
| Per 1-SD | 1.19 (1.14-1.25) | <0.001 | 1.12 (1.05-1.18) | <0.001 |
| Q1 | Ref |  | Ref |  |
| Q2 | 1.12 (0.98-1.28) | 0.098 | 1.17 (0.92-1.48) | 0.195 |
| Q3 | 1.19 (1.00-1.40) | 0.047 | 1.34 (1.02-1.77) | 0.036 |
| Q4 | 1.52 (1.32-1.75) | <0.001 | 1.57 (1.22-2.03) | <0.001 |
| *P_trend_* | <0.001 |  | <0.001 |  |
| **CRI-II-CRP** |  |  |  |  |
| Per 1-SD | 1.18 (1.12-1.24) | <0.001 | 1.10 (1.04-1.17) | 0.001 |
| Q1 | Ref |  | Ref |  |
| Q2 | 1.10 (0.96-1.25) | 0.159 | 1.08 (0.84-1.38) | 0.553 |
| Q3 | 1.17 (0.99-1.38) | 0.061 | 1.38 (1.04-1.83) | 0.024 |
| Q4 | 1.45 (1.26-1.66) | <0.001 | 1.43 (1.12-1.84) | 0.004 |
| *P_trend_* | <0.001 |  | <0.001 |  |
| **LCI-CRP** |  |  |  |  |
| Per 1-SD | 1.13 (1.07-1.19) | <0.001 | 1.09 (1.03-1.16) | 0.003 |
| Q1 | Ref |  | Ref |  |
| Q2 | 0.99 (0.87-1.12) | 0.866 | 0.90 (0.71-1.15) | 0.409 |
| Q3 | 1.09 (0.96-1.24) | 0.180 | 1.02 (0.77-1.35) | 0.908 |
| Q4 | 1.17 (1.03-1.34) | 0.020 | 1.25 (0.97-1.60) | 0.079 |
| *P_trend_* | 0.006 |  | 0.040 |  |
| **RC/HDL-C-CRP** |  |  |  |  |
| Per 1-SD | 1.17 (1.11-1.23) | <0.001 | 1.11 (1.04-1.18) | 0.002 |
| Q1 | Ref |  | Ref |  |
| Q2 | 0.96 (0.82-1.12) | 0.628 | 1.17 (0.92-1.49) | 0.198 |
| Q3 | 1.08 (0.95-1.24) | 0.244 | 1.34 (1.02-1.77) | 0.038 |
| Q4 | 1.29 (1.13-1.48) | <0.001 | 1.44 (1.11-1.87) | 0.007 |
| *P_trend_* | <0.001 |  | 0.005 |  |

Note: Models were adjusted for age, sex, marital status, education, smoking, drinking, diabetes, hypertension, BMI, and SBP.

Abbreviations: NHANES, National Health and Nutrition Examination Survey; CHARLS, China Health and Retirement Longitudinal Study; HR, hazard ratio; CI, confidence interval; Ref, reference.

# **Supplementary Table. 15** Sensitivity analyses excluding participants with known baseline use of lipid-lowering medications.

| **Parameters** | **NHANES** | | **CHARLS** | |
| --- | --- | --- | --- | --- |
|  | **HR (95% CI)** | ***P*** | **HR (95% CI)** | ***P*** |
| **non-HDL-C-CRP** |  |  |  |  |
| Per 1-SD | 1.22 (1.14-1.30) | <0.001 | 1.15 (1.09-1.22) | <0.001 |
| Q1 | Ref |  | Ref |  |
| Q2 | 1.03 (0.90-1.19) | 0.658 | 1.02 (0.81-1.29) | 0.852 |
| Q3 | 1.23 (1.01-1.50) | 0.036 | 1.34 (1.02-1.75) | 0.033 |
| Q4 | 1.39 (1.14-1.71) | 0.001 | 1.52 (1.21-1.90) | <0.001 |
| *P_trend_* | <0.001 |  | <0.001 |  |
| **RC-CRP** |  |  |  |  |
| Per 1-SD | 1.19 (1.12-1.27) | <0.001 | 1.15 (1.08-1.23) | <0.001 |
| Q1 | Ref |  | Ref |  |
| Q2 | 1.10 (0.91-1.33) | 0.318 | 1.12 (0.88-1.42) | 0.352 |
| Q3 | 1.06 (0.90-1.25) | 0.505 | 1.39 (1.09-1.77) | 0.007 |
| Q4 | 1.40 (1.17-1.68) | <0.001 | 1.59 (1.25-2.00) | <0.001 |
| *P_trend_* | <0.001 |  | <0.001 |  |
| **AIP-CRP** |  |  |  |  |
| Per 1-SD | 1.19 (1.11-1.27) | <0.001 | 1.13 (1.06-1.20) | <0.001 |
| Q1 | Ref |  | Ref |  |
| Q2 | 0.84 (0.71-1.01) | 0.066 | 0.89 (0.70-1.12) | 0.320 |
| Q3 | 0.90 (0.78-1.04) | 0.162 | 0.91 (0.71-1.17) | 0.462 |
| Q4 | 1.13 (0.95-1.35) | 0.159 | 1.17 (0.91-1.51) | 0.225 |
| *P_trend_* | 0.100 |  | 0.161 |  |
| **AC-CRP** |  |  |  |  |
| Per 1-SD | 1.22 (1.14-1.30) | <0.001 | 1.14 (1.07-1.21) | <0.001 |
| Q1 | Ref |  | Ref |  |
| Q2 | 1.09 (0.94-1.27) | 0.233 | 1.07 (0.84-1.37) | 0.575 |
| Q3 | 1.21 (1.02-1.43) | 0.027 | 1.22 (0.94-1.59) | 0.140 |
| Q4 | 1.43 (1.19-1.71) | <0.001 | 1.49 (1.18-1.86) | <0.001 |
| *P_trend_* | <0.001 |  | <0.001 |  |
| **CRI-I-CRP** |  |  |  |  |
| Per 1-SD | 1.23 (1.16-1.32) | <0.001 | 1.15 (1.09-1.22) | <0.001 |
| Q1 | Ref |  | Ref |  |
| Q2 | 1.13 (0.96-1.34) | 0.140 | 1.15 (0.92-1.44) | 0.208 |
| Q3 | 1.23 (1.02-1.48) | 0.027 | 1.27 (0.98-1.66) | 0.072 |
| Q4 | 1.51 (1.24-1.84) | <0.001 | 1.58 (1.27-1.97) | <0.001 |
| *P_trend_* | <0.001 |  | <0.001 |  |
| **CRI-II-CRP** |  |  |  |  |
| Per 1-SD | 1.22 (1.14-1.30) | <0.001 | 1.14 (1.07-1.20) | <0.001 |
| Q1 | Ref |  | Ref |  |
| Q2 | 1.07 (0.92-1.23) | 0.387 | 1.02 (0.80-1.31) | 0.871 |
| Q3 | 1.19 (1.00-1.42) | 0.049 | 1.29 (0.99-1.68) | 0.055 |
| Q4 | 1.39 (1.16-1.66) | <0.001 | 1.45 (1.16-1.81) | 0.001 |
| *P_trend_* | <0.001 |  | <0.001 |  |
| **LCI-CRP** |  |  |  |  |
| Per 1-SD | 1.16 (1.07-1.25) | <0.001 | 1.12 (1.06-1.19) | <0.001 |
| Q1 | Ref |  | Ref |  |
| Q2 | 1.00 (0.85-1.16) | 0.956 | 0.96 (0.76-1.22) | 0.742 |
| Q3 | 1.04 (0.91-1.19) | 0.568 | 1.10 (0.85-1.44) | 0.472 |
| Q4 | 1.14 (0.95-1.36) | 0.151 | 1.29 (1.03-1.62) | 0.030 |
| *P_trend_* | 0.115 |  | 0.013 |  |
| **RC/HDL-C-CRP** |  |  |  |  |
| Per 1-SD | 1.20 (1.12-1.28) | <0.001 | 1.14 (1.07-1.21) | <0.001 |
| Q1 | Ref |  | Ref |  |
| Q2 | 1.06 (0.89-1.26) | 0.532 | 1.12 (0.89-1.41) | 0.326 |
| Q3 | 1.09 (0.93-1.28) | 0.275 | 1.31 (1.00-1.71) | 0.046 |
| Q4 | 1.37 (1.16-1.62) | <0.001 | 1.46 (1.16-1.84) | 0.001 |
| *P_trend_* | <0.001 |  | <0.001 |  |

Note: Models were adjusted for age, sex, marital status, education, smoking, drinking, diabetes, hypertension, BMI, and SBP.

Abbreviations: NHANES, National Health and Nutrition Examination Survey; CHARLS, China Health and Retirement Longitudinal Study; HR, hazard ratio; CI, confidence interval; Ref, reference.

# **Supplementary Table. 16** Sensitivity analyses restricted to participants with complete data.

| **Parameters** | **NHANES** | | **CHARLS** | |
| --- | --- | --- | --- | --- |
|  | **HR (95% CI)** | ***P*** | **HR (95% CI)** | ***P*** |
| **non-HDL-C-CRP** |  |  |  |  |
| Per 1-SD | 1.23 (1.17-1.29) | <0.001 | 1.16 (1.09-1.22) | <0.001 |
| Q1 | Ref |  | Ref |  |
| Q2 | 1.09 (0.95-1.25) | 0.236 | 0.99 (0.77-1.27) | 0.915 |
| Q3 | 1.23 (1.04-1.46) | 0.016 | 1.28 (0.95-1.72) | 0.103 |
| Q4 | 1.55 (1.36-1.78) | <0.001 | 1.46 (1.12-1.91) | 0.006 |
| *P_trend_* | <0.001 |  | <0.001 |  |
| **RC-CRP** |  |  |  |  |
| Per 1-SD | 1.22 (1.16-1.28) | <0.001 | 1.16 (1.09-1.24) | <0.001 |
| Q1 | Ref |  | Ref |  |
| Q2 | 1.02 (0.85-1.23) | 0.816 | 1.08 (0.83-1.41) | 0.585 |
| Q3 | 1.14 (0.97-1.34) | 0.103 | 1.31 (0.98-1.74) | 0.064 |
| Q4 | 1.47 (1.28-1.70) | <0.001 | 1.57 (1.15-2.16) | 0.005 |
| *P_trend_* | <0.001 |  | 0.002 |  |
| **AIP-CRP** |  |  |  |  |
| Per 1-SD | 1.23 (1.17-1.30) | <0.001 | 1.13 (1.06-1.21) | <0.001 |
| Q1 | Ref |  | Ref |  |
| Q2 | 0.82 (0.71-0.95) | 0.010 | 0.86 (0.68-1.09) | 0.223 |
| Q3 | 0.92 (0.81-1.06) | 0.240 | 0.86 (0.64-1.15) | 0.314 |
| Q4 | 1.23 (1.06-1.42) | 0.006 | 1.15 (0.84-1.57) | 0.379 |
| *P_trend_* | <0.001 |  | 0.346 |  |
| **AC-CRP** |  |  |  |  |
| Per 1-SD | 1.25 (1.19-1.31) | <0.001 | 1.14 (1.07-1.21) | <0.001 |
| Q1 | Ref |  | Ref |  |
| Q2 | 1.02 (0.89-1.17) | 0.770 | 1.02 (0.79-1.31) | 0.905 |
| Q3 | 1.23 (1.04-1.45) | 0.016 | 1.12 (0.81-1.55) | 0.486 |
| Q4 | 1.50 (1.31-1.70) | <0.001 | 1.41 (1.03-1.93) | 0.032 |
| *P_trend_* | <0.001 |  | 0.019 |  |
| **CRI-I-CRP** |  |  |  |  |
| Per 1-SD | 1.26 (1.20-1.32) | <0.001 | 1.15 (1.08-1.22) | <0.001 |
| Q1 | Ref |  | Ref |  |
| Q2 | 1.10 (0.95-1.28) | 0.182 | 1.07 (0.84-1.36) | 0.574 |
| Q3 | 1.23 (1.02-1.48) | 0.028 | 1.19 (0.87-1.61) | 0.279 |
| Q4 | 1.60 (1.39-1.83) | <0.001 | 1.52 (1.13-2.05) | 0.006 |
| *P_trend_* | <0.001 |  | 0.004 |  |
| **CRI-II-CRP** |  |  |  |  |
| Per 1-SD | 1.24 (1.18-1.31) | <0.001 | 1.13 (1.07-1.20) | <0.001 |
| Q1 | Ref |  | Ref |  |
| Q2 | 1.07 (0.94-1.21) | 0.312 | 0.99 (0.77-1.27) | 0.936 |
| Q3 | 1.20 (1.00-1.43) | 0.047 | 1.22 (0.89-1.66) | 0.210 |
| Q4 | 1.50 (1.31-1.71) | <0.001 | 1.36 (1.02-1.82) | 0.038 |
| *P_trend_* | <0.001 |  | 0.015 |  |
| **LCI-CRP** |  |  |  |  |
| Per 1-SD | 1.19 (1.13-1.26) | <0.001 | 1.12 (1.05-1.19) | <0.001 |
| Q1 | Ref |  | Ref |  |
| Q2 | 0.97 (0.85-1.09) | 0.571 | 0.88 (0.69-1.13) | 0.328 |
| Q3 | 1.09 (0.95-1.24) | 0.224 | 0.90 (0.66-1.21) | 0.484 |
| Q4 | 1.24 (1.09-1.40) | <0.001 | 1.27 (0.94-1.72) | 0.117 |
| *P_trend_* | <0.001 |  | 0.095 |  |
| **RC/HDL-C-CRP** |  |  |  |  |
| Per 1-SD | 1.23 (1.17-1.29) | <0.001 | 1.15 (1.08-1.22) | <0.001 |
| Q1 | Ref |  | Ref |  |
| Q2 | 0.98 (0.83-1.15) | 0.799 | 1.05 (0.81-1.35) | 0.724 |
| Q3 | 1.08 (0.92-1.27) | 0.342 | 1.18 (0.88-1.59) | 0.268 |
| Q4 | 1.38 (1.21-1.59) | <0.001 | 1.43 (1.03-1.98) | 0.032 |
| *P_trend_* | <0.001 |  | 0.023 |  |

Note: Models were adjusted for age, sex, marital status, education, smoking, drinking, diabetes, hypertension, BMI, and SBP.

Abbreviations: NHANES, National Health and Nutrition Examination Survey; CHARLS, China Health and Retirement Longitudinal Study; HR, hazard ratio; CI, confidence interval; Ref, reference.

# **Supplementary Figure. 1** Analysis of variance inflation factors and Spearman correlation coefficients for the variables included in NHANES.

Abbreviations: NHANES, National Health and Nutrition Examination Survey; BMI, body mass index; SBP, systolic blood pressure.

# **Supplementary Figure. 2** Analysis of variance inflation factors and Spearman correlation coefficients for the variables included in CHARLS.

Abbreviations: CHARLS, China Health and Retirement Longitudinal Study; BMI, body mass index; SBP, systolic blood pressure.

# **Supplementary Figure. 3** Event distribution during the follow-up period.

Abbreviations: NHANES, National Health and Nutrition Examination Survey; CHARLS, China Health and Retirement Longitudinal Study.

# **Supplementary Figure. 4** Weighted Kaplan-Meier survival curves by quartiles of non-traditional lipid-inflammatory parameters in NHANES.

Abbreviations: NHANES, National Health and Nutrition Examination Survey.

# **Supplementary Figure. 5** Weighted Kaplan-Meier survival curves by quartiles of non-traditional lipid-inflammatory parameters in CHARLS.

Abbreviations: CHARLS, China Health and Retirement Longitudinal Study.

# **Supplementary Figure. 6** Weighted RCS analyses of the non-traditional lipid-inflammatory parameters with all-cause mortality in CHARLS.

Note: Models were adjusted for age, sex, marital status, education, smoking, drinking, diabetes, hypertension, BMI, and SBP.

Abbreviations: CHARLS, China Health and Retirement Longitudinal Study.

# **Supplementary Figure. 7** Calibration curves for Cox proportional hazards models to predict all-cause mortality at different follow-up time points.

Note: Base model included age, sex, marital status, education, smoking, drinking, diabetes, hypertension, BMI, and SBP.

Abbreviations: NHANES, National Health and Nutrition Examination Survey; CHARLS, China Health and Retirement Longitudinal Study.
